# Population-level implications of the Israeli booster campaign to curtail COVID-19 resurgence

**DOI:** 10.1126/scitranslmed.abn9836

**Published:** 2022-04-12

**Authors:** Nir Gavish, Rami Yaari, Amit Huppert, Guy Katriel

**Affiliations:** ^1^ Faculty of Mathematics, Technion Israel Institute of Technology, Haifa, 32000, Israel; ^2^ The Bio-statistical and Bio-mathematical Unit, The Gertner Institute for Epidemiology & Health Policy Research, Sheba Medical Center, 52621, Israel; ^3^ School of Public Health, Tel Aviv University, 6997801, Israel; ^4^ Department of Applied Mathematics, ORT Braude College of Engineering, Karmiel, 2161002, Israel

## Abstract

Israel was one of the first countries to administer mass vaccination. Consequently, it was among the first countries to experience substantial breakthrough infections due to the waning of vaccine-induced immunity, which led to a resurgence of the epidemic. In response, Israel launched a booster campaign to mitigate the outbreak, and was the first country to do so. Israel’s success in curtailing the Delta resurgence while imposing only mild non-pharmaceutical interventions influenced the decision of many countries to initiate a booster campaign. By constructing a detailed mathematical model and calibrating it to the Israeli data, we extend the understanding of the impact of the booster campaign from the individual to the population level. We used the calibrated model to explore counterfactual scenarios in which the booster vaccination campaign is altered by changing the eligibility criteria or the start time of the campaign and to assess the direct and indirect effects in the different scenarios. The results point to the vast benefits of vaccinating younger age groups that are not at a high risk of developing severe disease but play an important role in transmission. We further show that when the epidemic is exponentially growing the success of the booster campaign is highly sensitive to the timing of its initiation. Hence a rapid response is an important factor in reducing disease burden using booster vaccination.

## Teaser

A mathematical model fit to detailed data analyzes the impact of the Israeli booster campaign and possible alternative policies.

## INTRODUCTION

During June 2021, Israel experienced an exponential rise in COVID-19 cases, with many infections and severe cases reported among vaccinated individuals. At that stage, roughly 80% of the eligible population and two-thirds of the entire population were vaccinated, following a successful campaign using two doses of the BNT162b2 vaccine (*1*–*3*). Initially, it was unclear to what extent resurgence was driven by increased infectiousness of the Delta variant, by heightened immune evasion, or by the waning of vaccine-elicited immunity. A nationwide study in Israel reduced the uncertainty by demonstrating a strong effect of waning immunity in all age groups six months from vaccination (*4*). Israel faced a dilemma regarding the administration of booster vaccinations because, at that stage, the BNT162b2 booster vaccination had not yet been approved by the United States Food and Drug Administration (FDA) or any other regulatory agency.

To curtail the Delta outbreak, Israel began administering booster vaccinations on July 30, 2021. Initially, booster vaccinations were restricted to ages 60 and older, but eligibility was rapidly extended to other age groups so that by the end of August 2021, individuals of age 16 and older who were at least five months past their second dose were eligible for booster vaccination. During August, ∼2.25 million booster vaccinations were administered, and by December 2021, an overall of ∼4 million individuals (∼80% of the eligible population) received the booster.

The example of Israel’s success in curtailing the Delta resurgence without a lockdown and with mild non-pharmaceutical interventions has provided empirical evidence that assisted the US FDA in the approval process of the third dose and influenced the decision of many nations to initiate a booster campaign. There is, however, an ongoing debate and uncertainty regarding the required extent of a booster campaign, for example as to which age groups should be boosted, and the importance of a rapid vaccination effort.

In this work, we present an in-depth analysis of the population-level impact of the different elements of the booster campaign on epidemic outcomes. We developed a transmission model that incorporates the waning of vaccine-induced immunity and its buildup after boosting. The model accounts for vaccine and booster administration per age group at a daily resolution and is calibrated using real-world data from Israel in the period from July 1^st^ to November 25^th^ 2021. The model was fitted to time series of polymerase chain reaction (PCR)-confirmed cases in 10-year age groups and different vaccination states. We used the calibrated model to study the impact of the booster campaign by quantifying the outcomes of counter-factual scenarios such as the application of alternative boosting campaigns in which boosting is restricted to specific age groups or in which the timing of the booster campaign is modified.

Direct protection afforded to those vaccinated is often estimated by conducting clinical trials and observational studies comparing outcomes in vaccinated and unvaccinated individuals (*4*, *5*). However, the indirect protection provided by reducing transmission at the population level is a key consideration when evaluating vaccination policies. We used the model to disentangle indirect effects by comparing outcomes of the vaccination campaign to expected outcomes in the absence of vaccinations. By modelling the ‘competition’ between the vaccination campaign and the spread of the epidemic, this study thus demonstrates the contribution of mathematical modelling to analyzing data and obtaining a retrospective understanding of the role of the different mechanisms in generating the observations. Such an understanding is crucial for making future projections and planning interventions.

## RESULTS

### Capturing the dynamics of the Delta outbreak

**Fig. 1.**
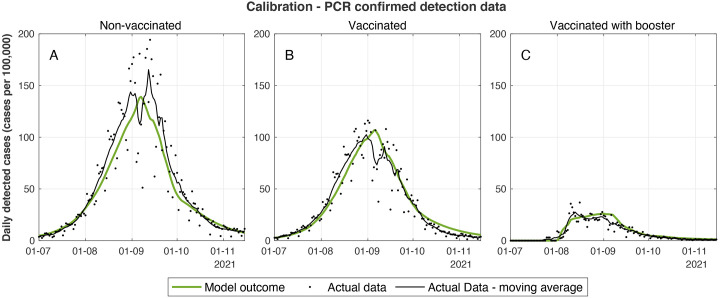
**Calibrated model outcomes vs actual case data. (A-C)** Daily number of new detected cases per 100,000 people who were not vaccinated (A), who received two vaccine doses (B), and who received three vaccine doses (C) showing both seven-day moving average of observed data (black markers) and model outcome (green curves). The data series presented in these graphs are aggregated from 27 data series that were used for model calibration. Graphs corresponding to each of the data series are presented in the supplementary materials.

We developed a model calibrated by real-world data of Israel during the Delta surge that captured the dynamics well, both before and during the period of the booster vaccination campaign (**Fig. 1**). The model relies on eight fitted parameters (see materials and methods and supplementary materials for details.) All estimated parameter values (**table S1**) were within reasonable ranges of epidemiological values, which further supports the appropriateness of the modeling scheme. We note that during September, the epidemiological data displayed some variations and irregularities primarily due to a sequence of Jewish holidays that affected the degree of the testing effort as well as patterns of transmission.

**Fig. 2.**
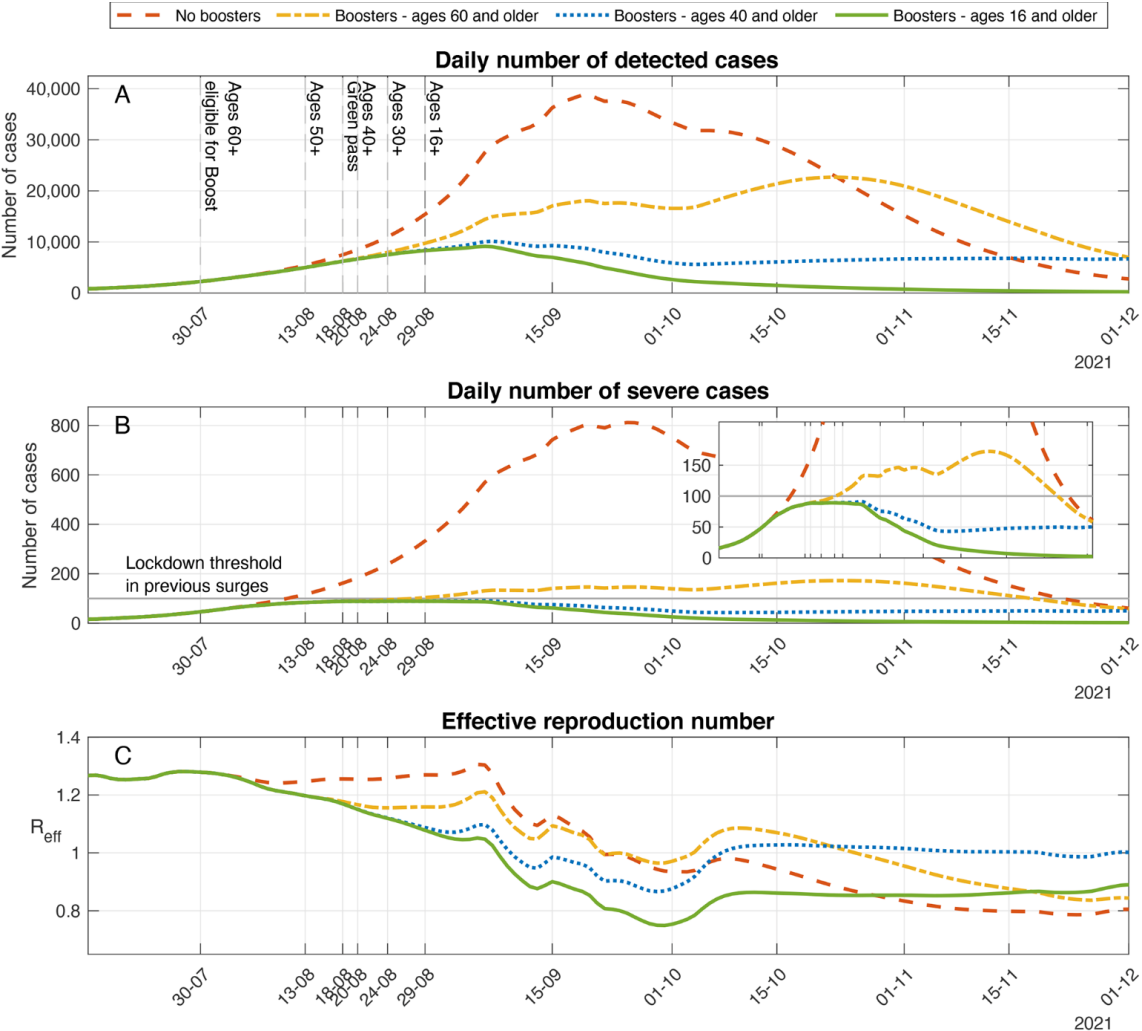
Effect of the booster campaign on detected cases, severe cases, and the effective reproductive number. Model projections for epidemic spread in counterfactual scenarios in which no boosters are administrated (dashed curve), boosters are administrated only to individuals of age 60 and older (dash-dotted curve), boosters are administered only to individuals of age 40 and older (dotted curve), as well as a scenario that accounts for the actual numbers of boosters that were administered until the end of November 2021 (solid curve). **(A)** Daily number of detected cases. **(B)** Daily number of severe cases. Inset graph presents the same data as in B, but with lower values of the vertical axis. **(C)** Effective reproductive number.

After calibration of the model, we first used it to explore a scenario in which no booster vaccinations are administered, and no additional non-pharmaceutical interventions are implemented. In the absence of boosters, the model projected the continued rise of a substantial outbreak reaching a peak of ∼38,800 cases detected daily and ∼810 new severe cases per day, with 64 of the infections occurring among individuals who had received two doses of vaccine (red dashed curve, Fig. 2A,B). These numbers of severe cases are far beyond the threshold at which medical care in Israel can be provided without being severely compromised. Indeed, prior to the Delta surge, Israel reached a peak of roughly 190 new severe cases hospitalized per day. Such patient loads in hospitals had been estimated to have resulted in as much as 25% excess in-hospital mortality in Israel (*6*). In fact, during previous surges, severe non-pharmaceutical interventions were applied at much lower rates of severe cases. For example, Israel entered a lockdown on September 18^th^, 2020 with ∼80 new severe cases per day and an additional lockdown on December 27^th^, 2020 with ∼100 new severe cases per day.

The assessment of the situation during July indicated the urgent need of applying non-pharmaceutical interventions or other restrictions to prevent catastrophic outcomes. In parallel, intensive research efforts led to the understanding that the waning of vaccine efficacy in Israel was a dominant factor in the resurgence of the epidemic spread (*4*). Additionally, using the same modeling framework presented in this work, our quantitative assessment of the population-level effects of administrating a booster vaccine to the vaccinated population, particularly those vaccinated at the initial stages of the Israeli vaccination campaign, showed benefits in most scenarios tested, with a sharp reduction in the number of cases and the burden of hospitalizations. Following these studies and the recommendation of the pandemic advisory committee, the Israeli ministry of health decided to launch a booster vaccination campaign.

### Curtailing the outbreak using boosters

On July 12^th^, 2021, Israeli health authorities recommended the administration of a third dose to high-risk populations. The official booster campaign was initiated on July 30^st^ with a recommendation to vaccinate individuals age 60 and older who had received a second Pfizer BNT162b2 vaccine dose at least five months before. The age of eligibility was gradually extended during August: ages 50-59 on August 13; ages 40-49, August 20; ages 30-39 August 24, and ages 16 and older on August 29. During the campaign, ∼42% of the population or ∼4 million individuals were given a booster vaccination (see (*5*) for details). To assess the population-level effect of the actual booster campaign in Israel, we relied on the model calibrated with data from the Delta surge in Israel and compared the model outcomes to model projections in a ‘hypothetical’ scenario in which no booster vaccinations are administered. Comparison of the number of cases under no booster versus booster for ages 16+ scenarios demonstrates the impact of the booster campaign in curtailing the outbreak (Fig. 2, A-C). In particular, we found that the overall number of cases in the simulation that accounts for booster uptake was 80.4% (CI; 78.1%-82.0%) smaller than the number of cases projected during the resurgent wave in the absence of boosters or other interventions. Similarly, we estimated that booster vaccination reduced mortality by 90.8% (CI; 91.0%-91.6%).

### Assessing different boosting policies

Israel decided to administer the booster to the general population rather than concentrate on the elderly (ages 60 and older) and other high-risk groups. There is an ongoing debate as to whether such a policy is best suited when the primary goal is to reduce the load on the health care system. The uncertainty concerns the magnitude of reduction in severe disease cases due to decreases in transmission in low-risk populations. To estimate the impact of boosting low-risk groups, we considered counter-factual scenarios in which the booster vaccination campaign is modified so that smaller segments of the population are boosted. Specifically, we examined cases in which booster administration is restricted to ages 60 and older or to ages 40 and older. To do so, we repeated the simulation presented in Fig. 2 while “removing” all booster shots given to individuals under the age of 60 or 40, respectively, and leaving the rates of vaccination in the eligible groups unchanged.

In the case that booster vaccines are given only to age groups 60 and older, we observed that the outbreak would be considerably reduced compared to a case in which no boosters are given (dash-dotted yellow curves, Fig. 2A,B). Roughly a third of these averted cases are of ages below 60, demonstrating strong indirect effects of booster vaccination (see below for a quantitative assessment of the indirect effect of the booster). The outbreak, however, is not reduced to the same extent as in the case that boosters are provided to all eligible ages (green solid curve). Indeed, we observed that, under the conditions considered, restricting booster eligibility to ages 60 and older led to an increase of 397%, 299%, and 272% in the number of confirmed, severe cases, and deaths respectively, relative to the number of cases when all individuals of age 16 and older are provided a booster dose (Table 1). As expected, when boosters are restricted to age groups 40 and older, the outcomes are closer to those attained when boosters are given to ages 16 and older. Nevertheless, there is an increase of 225%, 177%, and 174% in the number of confirmed, severe cases, and deaths, respectively (Table 1 and dotted blue curves in Fig. 2A,B). An important difference between the outcomes is that in the case boosters are given to ages 40 and older, the epidemic slowly decays until it stagnates with an effective reproduction number of roughly one (green solid curve in Fig. 2C). This stagnation leads to a projected average of 6,700 confirmed cases and 50 new severe cases per day in late November. In comparison, when boosters are given to ages 16 and older, the decay of the epidemic is faster with an effective reproduction number of ∼0.86, leading to 200-250 confirmed cases and 2-3 new severe cases per day in late November (Fig. 2C). Consequently, when the booster campaign curtails the epidemic spread, the risk of a resurgence due to a new variant, seasonality, national holidays, or future waning can increase when booster eligibility is restricted to a smaller population. The reason is that fewer people gain protection through vaccination or infection.

**Table 1.** Change in epidemiological outcomes in various booster policies relative to the outcomes of the actual booster campaign. Values in parentheses correspond to the changes in epidemic outcomes attained by using the parameter values at the boundary of the 95% confidence interval of parameter estimates. Early and late schedules refer to scenarios in which the booster campaign is advanced or delayed by two weeks, respectively.

### Timing of the boosting campaign

The Israeli Ministry of Health debated the need for a booster campaign during the entire month of July. In early July, it was unclear to what extent the resurgence was driven by increased infectiousness of the Delta variant, by heightened immune evasion, or by the waning of vaccine-elicited immunity. Accordingly, the potential effectiveness of administering boosters in curtailing the outbreak was also unclear. The picture was partially clarified in mid-July, as it became evident that waning vaccine immunity played a crucial role in driving the outbreak (*4*). Israeli authorities decided to initiate an extensive booster campaign within two weeks of this observation. It is therefore interesting to evaluate potential outcomes in case the booster campaign had been initiated at an earlier date, as well as the consequences had the decision to initiate the campaign been somewhat delayed. We addressed these questions by running the model simulation with the calibrated parameters, changing only the starting date of the vaccination campaign.

**Fig. 3.**
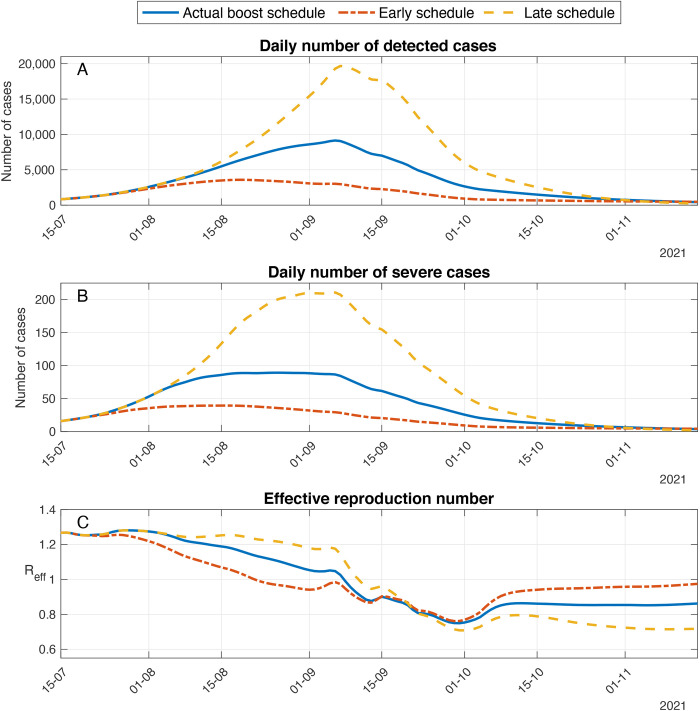
Effect of timing of the start date of the booster campaign on epidemic outcomes. Model projections for epidemic spread in a scenario in which the booster campaign is delayed (dashed curve) or advanced by two weeks (dash-dotted curve) compared to a scenario that accounts for the actual timing of the booster campaign (solid curve). **(A)** Daily number of detected cases. **(B)** Daily number of severe cases. **(C)** Effective reproductive number. **Table:** 

Delaying or advancing the initiation of the vaccination campaign by two weeks leads to major differences in the outcomes of the booster campaign (Fig. 3A,B). As expected, delaying the booster vaccination campaign by two weeks results in a larger outbreak (see for comparison the differences between the solid blue line and the dashed yellow curve in Fig. 3). In this case, we found that the peak number of severe cases and mortality are comparable to those attained when boosters are restricted to ages 60 and above. In the reverse case, in which the booster vaccination campaign is advanced by two weeks, the epidemic spread is curtailed at an early stage. The early epidemic decay leads to a reduction of 53%, 51% and 50% in the number of confirmed, severe cases and deaths, respectively, relative to the numbers attained with the actual booster schedule (see the differences between the solid blue line and the dash dotted red curve in Fig. 3A,B and Table 1). In this case, however, the epidemic decay is very slow. Eventually, by mid-October it stagnates with an effective reproduction number slightly below one (Fig. 3C). Accordingly, the projected number of cases in late November is 400 confirmed cases and 4 new severe cases daily. In comparison, the model projections corresponding to the actual booster campaign give rise to roughly half the number of confirmed and severe daily cases in the same period. Consequently, we observed that whereas early administration of booster vaccines efficiently curtails the epidemic wave in the short term, it increases the risk of resurgence in the longer run due to the fact that fewer recovered individuals are accumulated.

**Booster Policy**	**Change in number of cases**	**Change in number of severe cases**	**Change in mortality**
No boost	501% (457-556)	904% (839-990)	1086% (1009-1189)
Age 60 and above	397% (361-443)	299% (275-330)	272% (251-300)
Age 40 and above	225% (208-245)	177% (166-191)	174% (164-189)
Early schedule	53% (52-54)	51% (51-52)	50% (50-51)
Late schedule	171% (164-178)	182% (176-189)	188% (182-195)

### Quantification of direct and indirect effects

Indirect benefits are a crucial consideration when evaluating SARS-CoV-2 vaccination policies (*7*), but disentangling indirect effects from numerous types of epidemic field observations is challenging (*8*). Here, we used simulations (*9*) to explicitly quantify the indirect protective effect of the booster vaccination campaign due to reduction of transmission. To do so, we used the model to compute epidemic outcomes in a scenario in which booster vaccination is regarded as non-protective. In such a case, the direct effect of booster vaccination is measured as the number of infections among those who received the booster, because these infections would not have occurred if the booster vaccine were fully protective. The indirect effect is computed as the difference between the total excess infections that would have occurred if boosters were not administered and the number of infections prevented by a direct protective effect (*9*). Our computation shows that among the cases reduced due to the booster campaign, ∼54% were reduced due to direct protection, whereas the rest were due to indirect protection. We further found that ∼73% of the reduction in new daily severe cases was due to direct protection, while the rest were due to indirect protection. Note that the above computation does not take into account the occurrence of breakthrough infections among those who received a booster. The very low rate of such breakthrough infections in the relevant period justifies this approximation. Accounting for breakthrough infections in the computation would give slightly more weight to indirect protection.

The above results quantify the considerable impact of booster vaccination in reducing transmission and providing indirect protection to those susceptible to infection. These results point to the vast benefits of vaccinating younger age groups that are not at a high risk of developing severe disease but that do play an important role in transmission.

## DISCUSSION

Israel was one of the first countries to administer mass vaccination and executed it with high roll-out rates. Due to early vaccination, Israel was also among the first countries where the waning of vaccine protection occurred. Identification of the nature of vaccine waning (*4*) led Israeli authorities to initiate an extensive booster campaign that curtailed the Delta resurgence. Previous studies have quantified the effects of boosting on an individual level (*5*, *10*, *11*). Here, we expand our understanding to the population level by constructing a mathematical model that accurately describes the Delta surge dynamics in Israel. The modeling framework allowed us to compare alternative boosting policies to ascertain the trade-offs of vaccinating different age groups and the importance of an early and extensive response. In addition, the mathematical model allowed exploration of the relative role of direct and indirect protection on different outcomes of the disease.

Our results indicate that, if a booster campaign had not been undertaken, Israel would have needed to apply extensive non-pharmaceutical interventions to prevent a destructive epidemic wave. To quantify the potential severity of the Delta outbreak, we used a counterfactual assumption that boosters had not been provided. The model estimated that without boosting or non-pharmaceutical interventions, the potential of the Delta wave was an increase of 1086% in mortality, of 904% in severe disease, and a 501% increase in detected infections relative to actual outcomes. Overall, providing a booster vaccination to roughly 40% of the population led to a ∼80% decrease in the number of cases. The gap between these numbers demonstrates the substantial indirect protection of the booster vaccination.

Quantifying the extent of direct and indirect protection is crucial in building strategies to cope with the pandemic or end it (*7*). The direct protection of the booster has been estimated in both clinical trials and observational studies (*5*, *12*, *13*). Indirect protection due to parental vaccination was estimated in household studies (*10*, *11*). Similarly, the household study (*14*) found that the odds ratio of a secondary case in a vaccinated individual was 0.54 of an unvaccinated individual. However, the full extent of the indirect protection by reducing transmission cannot be fully estimated in the field. Mechanistic modelling can help bridge this gap. Indeed, we demonstrate the use of the model to explicitly quantify the indirect protective effect of the booster vaccination campaign by studying appropriate counterfactual scenarios.

In addition, we assess the expected impact of more restricted booster vaccination campaigns and compare alternative policies. Booster availability restricted to age groups 40+ or 60+ would have significantly reduced infection, severe disease, and deaths. However, it is far less effective, with respect to all outcomes, than boosting wider segments of the population. In the case of limited vaccine supply, these results support the consideration of vaccination strategies involving partial doses designed to extend vaccine coverage (*15*, *16*). We further show that when the epidemic is exponentially growing, the success of the booster campaign is sensitive to the timing of the initiation. For instance, advancing the campaign two weeks earlier would have led to an overall decrease in the number of cases by roughly a factor of three compared to the actual booster campaign. This sensitivity to the timing manifests the competition between the epidemic spread and the booster roll-out rate and the fact that booster vaccinations provide protection to an individual within a short period from receiving the vaccination.

This study is subject to several limitations. Model parameters such as the probability of detection of infections or the probability of developing severe outcomes were assumed to be fixed in time. These parameters, however, may vary in time, for example the probability of an infection being detected may decrease as testing capacity is approaches its maximal capacity or due to changes in the screening policy in the school system. Particularly, the period under study included both the school summer vacation during July-August and Jewish holidays during September. These events also led to differences in transmission patterns among the different sectors in Israel, for example the education system in the Arab sector operated continuously throughout September, unaffected by the Jewish holidays. Such sector-level details were not captured by the model. Last, the model does not capture possible behavioral responses. For example, it could be that as incidence of infection increases, people are not only interacting less frequently - an effect captured by the mobility factors - but are also being more cautious when interacting with others. In the reverse direction, people may become less cautious if they believe they are protected by a vaccine.

Additional limitations stem from the fact that this work aims at understanding the population-level effect of booster vaccine protection in the short-term. Accordingly, our modelling neglects several factors that are likely to impact epidemic outcomes in the longer term, on a time scale of years. In particular, we did not account for demographic turnover (births, deaths, and aging), waning of convalescent immunity, or seasonality in the transmission rate.

We have used the data and parameter values representing the population’s epidemiology and behavior during the Delta resurgence in Israel to calibrate the model. The focus of this case study raises a natural question regarding the extent to which our conclusions can be generalized to other countries. Transmission dynamics are sensitive to the social contact patterns. The social contact matrix used in the model is composed of a time-varying linear combination of contact matrices in key social settings (*8*) that was determined using Google’s COVID-19 community mobility data. We found that the number of social contacts of those above the age of 60 during the Delta outbreak in Israel was relatively high compared to previous waves and the number of social contacts described by the commonly used POLYMOD contact matrix in this age group. Indeed, the contribution of age group 60 and older to the basic reproduction number in the time-varying social contact matrix used in the simulation varied within 9-11%, whereas the contribution of age group 60 and older to the basic reproduction number in the POLYMOD contact matrix adapted to Israel (*17*) is just 2%. We also note that the impact of extending eligibility for the booster vaccine to younger age groups also crucially depends on the vaccine uptake rates, which was lower in younger age groups. The above considerations warrant care in adapting the results to other countries.

Because this study focused on the period in which the circulating variant was the Delta variant, it is natural to ask whether our conclusions can be generalized to other variants, and in particular to the Omicron variant which emerged in the months following the period studied here. Although we expect the general qualitative conclusions regarding the impact of booster vaccination, the importance of indirect protection effects, and the significance of timing to hold true in many scenarios, there are several features of the Omicron variant which would require modification of the modelling and should lead to changes in quantitative outcomes. First, the Omicron resurgence was driven by vaccine breakthrough rather than vaccine waning. Second, a fourth dose of vaccine, as was offered in Israel to individuals of ages 60 and over, has been shown to be considerably less effective in increasing protection against infection and severe outcomes relative to the third dose (*18*). Additionally, reinfections are much more common during the Omicron surge. Last, the Omicron variant is less virulent than previous variants, leading to a lower rate of severe cases among those infected. Obtaining a refined picture of the impact of a booster vaccination in the case of an outbreak of such different nature requires adaptation of the model and parameters to account for changes in transmissibility, vaccine and booster efficacy, waning profile, and the possibility of reinfections. The framework developed in the course of this study is sufficiently flexible to allow such modifications - a detailed analysis considering various scenarios will be presented elsewhere.

To conclude, this study highlights the importance of using boosters to curtail outbreaks resulting from waning immunity. The modeling demonstrates that both a rapid response, and one that includes the sub- populations which play a significant role in transmission, even if they are at low risk of severe disease, are significant for reducing the disease burden. As the world faces the danger of new variants of concern it is crucial to improve our ability to minimize the toll of the disease.

## MATERIALS AND METHODS

### Study design

To capture the transmission dynamics of the Israeli fourth wave from July 1^st^ to November 25^th^ 2021, we developed a discrete-time age- of-infection age-stratified transmission model that includes vaccination and booster administration and takes account of the waning of vaccine-induced immunity depending on time since vaccination. To calibrate and validate the model, we used nationwide data from Israel consisting of incidence of detected infections and of severe disease, as well as of vaccination rates stratified according to age and at daily resolution. Additional data used consisted of Google mobility data and school vacation schedules. Estimates of vaccine waning rates from other studies carried out in Israel (*4*, *5*) were used for parameterizing the model. Eight model parameters were estimated by fitting the model to the incidence data using maximum likelihood, with 95% confidence intervals for these parameters generated using a likelihood profile approach. The calibrated model was used to examine counterfactual scenarios involving changes in the booster campaign.

A complete account of the data, model, and methods used is provided in the supplementary material. Here we provide a brief description of the main features. The study was approved by the Institutional Review Board of the Sheba Medical Center. Helsinki approval number: SMC-8228-21.

**Data:** Data was extracted from the Israel Ministry of Health’s database. The information per individual consisted of age, place of residency, dates of PCR tests, vaccination dates (first, second, and third doses), severe COVID-19-related hospitalization, and mortality. Severe disease was defined according to the US National Institutes of Health COVID-19 treatment guidelines (*19*). The incidence data sets include all PCR confirmed (detected) and severe Covid-19 cases in Israel from July 1^st^ to November 25^th^ 2021. The data sets were stratified by age-group (9 age-groups: 0-9,10- 19,20-29,30-39,40-49,50-59,60-69,70-79,80+) and by vaccination status: unvaccinated, vaccinated with two doses and booster-vaccinated. Overall, 496,625 cases were detected during this time period: 5,025 of these cases (1%) did not have an age indication and were removed. Of the 491,600 detected cases remaining, 5,743 (1.2%) became severely ill at some point after detection.

**Waning rates:** We relied on published estimates of the waning rate of vaccine protection from infection (*4*, *20*) to estimate the loss in protection as a function of time since vaccination. As for protection from severe disease beyond the protection against infection, we assumed that the probability of a vaccinated individual to develop severe symptoms, conditional upon being infected, did not change with time. This implies that the waning rate of vaccine protection from severe outcomes was determined by the waning rate of vaccine protection from infection. This assumption is supported using estimates in (*4*).

In the absence of data supporting quantitative estimates, we assumed that the waning rate of booster protection from infection and severe outcomes was half the waning rate of second dose vaccine protection. We note that our results are insensitive to this assumption because booster efficacy remained high during the time span of the present study.

**Reinfections:** During the period of study, reinfections constituted roughly 1% of confirmed cases. Accordingly, we neglected the possibility of reinfections in the model.

**Transmission model:** We developed a discrete-time age-of-infection age-stratified transmission model. The infection process was modeled using a social contact matrix that determines the average number of daily interactions, for example, the number of contacts a 53-year-old has with individuals in the 30-39 age group. The social contact matrix was composed of a linear combination of contact matrices customized to Israel in key social settings (*21*): household, work, community, and school. The time-dependent relative weight of the school matrix was set according to school vacations, whereas the relative weights of the other three matrices were determined using Google’s COVID-19 community mobility data (*22*). Additional parameters were inferred by fitting the model to data collected during the period from July 1^st^ to November 25^th^ 2021, when the Delta surge took place in Israel.

**Observation model and parameter inference:** The observation model connects the infection counts generated by the epidemic model to those observed in the data, taking into account the delay from infection to the identification of a case and the fact that only a fraction of infections are identified. Specifically, we considered time series of confirmed cases stratified by 10-year age groups and vaccination status. These amounted to an overall 27 time series at daily resolution.

We used maximum likelihood to estimate model parameters, where the likelihood function assumes a negative binomial distribution of the observed data, with its mean given by the product of the number of infections provided by the transmission model and the detection rate. Overall, eight parameters were estimated: the reproductive number in the beginning of July; three parameters describing the intensity of contacts in workplaces, schools, and the community relative to household contacts; a parameter corresponding to the relative susceptibility of children; the dispersion of the negative binomial used in the observation model; and two parameters related to the age-dependent detection rate of infected individuals.

We verified that simulations of the calibrated model agreed with the time series of severe cases stratified according to age and vaccination status, as well as to the detected case data to which the model was fitted, at daily resolution. We used a likelihood profile approach to compute 95% confidence intervals (CI) for the parameter estimates. The CI for each parameter was taken to be the range of parameter values in which the log-likelihood values are within 1.92 from the maximum-likelihood estimate.

## Supplementary Materials

### This PDF file includes:

Materials and methods Figs. S1 to S13 Tables S1 and S2 References (*23*, *24*)

### Other Supplementary Material for this manuscript includes the following:

MDAR Reproducibility Checklist
