# Supplementary material for: Population-level implications of the Israeli booster campaign to curtail COVID-19 resurgence
Source: Sci Transl Med. 2022 Apr 12:eabn9836. doi: 10.1126/scitranslmed.abn9836 (PMC9012104; doi:10.1126/scitranslmed.abn9836)
Supplement: Supplementary file 1 — Materials and methods Figs. S1 to S13 Tables S1 and S2 References ( 23 , 24 ) [file scitranslmed.abn9836_sm.pdf]

Science Translational Medicine
AAAS

Supplementary Materials for

Population-level implications of the Israeli booster campaign to curtail COVID-19 resurgence

Nir Gavish *et al.*

Corresponding author: Nir Gavish, ngavish@technion.ac.il

DOI: 10.1126/scitranslmed.abn9836

The PDF file includes:

Materials and methods
Figs. S1 to S13
Tables S1 and S2
References (*23*, *24*)

Other Supplementary Material for this manuscript includes the following:

MDAR Reproducibility Checklist
Data files S1 and S2

Supplementary information

Booster vaccination to curtail COVID-19 resurgence - population-level implications of the Israeli campaign

Nir Gavish[1*†], Rami Yaari[2†], Amit Huppert[2,3], Guy Katriel[3]

[1]Faculty of Mathematics, Technion Israel Institute of Technology, Haifa, 32000, Israel

[2]The Bio-statistical and Bio-mathematical Unit, The Gertner Institute for Epidemiology & Health Policy Research, Sheba Medical Center, 52621, Israel

[3]School of Public Health, Tel Aviv University, 6997801, Israel

[4]Department of Applied Mathematics, ORT Braude College of Engineering, Karmiel, 2161002, Israel

[*]To whom correspondence should be addressed; E-mail: ngavish@technion.ac.il

[†]These authors contributed equally to this work

Materials and Methods

Data sources

The incidence data sets include all PCR-confirmed (detected) and severe Covid-19 cases in Israel from July 1st to November 25th 2021. The data sets are stratified by age-group (9 age-groups: 0-9,10- 19,20-29,30-39,40-49,50-59,60-69,70-79,80+) and by vaccination status: unvaccinated, vaccinated with two doses and booster-vaccinated. Overall, 496,625 cases were detected during this time period: 5,025 of these cases (1%) did not have an age indication and were removed. Of the 491,600 detected cases remaining, 5,743 (1.2%) became severely ill at some point after detection.

The data files are available at github.com/NGavish/boosterVaccinationModel.git.

Note: Data cells with non-zero values smaller than 5 were randomized to comply with the Israeli Ministry's of Health regulations.

Transmission model

We develop a discrete-time age-of-infection model (also known as a renewal equation-type model). Accounting for age of infection enabled us to take into account the appropriate generation-time distribution. Additionally, this approach enabled us to precisely incorporate the processes of primary and booster vaccination, based on the detailed data at daily resolution of the vaccination schedule and the increase of susceptibility of those vaccinated with time since vaccination, as estimated in previous studies from Israel.

The source code of the transmission model is available at github.com/NGavish/boosterVaccinationModel.git. In what follows, we describe the components and structure of the model.

Dynamic variables

The population is divided into n = 9 age groups of sizes $N_j (1 \leq j \leq n)$. The discrete variable t refers to time in days, where t = 0 is the first day on which vaccinations were administered in Israel (Dec. 20, 2020). We distinguish between non-vaccinated individuals (nv), vaccinated (v) and those vaccinated with three doses (b), and compute the evolution of the following variables:

- $S(t, j)$ is the overall number of susceptible, non-vaccinated individuals of age group j on day t.
- $V(t, j, s)$ is the overall number of individuals of age group j who had been vaccinated s days before day t, and have not been infected up to day t.
- $B(t, j, s)$ is the overall number of individuals of age group j, who have received a booster dose s days before day t, and have not been infected up to day t.
- $i_{nv}(t,j)$ is the number of newly infected individuals of age group j on day t who have never been vaccinated.
- $i_v(t, j, s)$ is the number of newly infected individuals of age group j on day t who were vaccinated s days before day t.
- $i_b(t, j, s)$ is the number of newly infected individuals of age group j who received a booster vaccination s days before day t.

Model equations

The daily incidence of infections of unvaccinated individuals in age group *j* on day *t* is given by

$$inv(t,j) = S(t,j)Fj(t) \tag{S1a}$$

where Fj (t) is the force of infection, that is the daily probability of an unvaccinated individual from age group j becoming infected. The force of infection is given by

$$F_i(t) = \sum_{j=1}^{n} \frac{\beta_{ij}(t)}{N_j} \sum_{\tau=1}^{\tau_{max}} P_\tau \cdot [i_{nv}(t-\tau,j) + \hat{\iota_v}(t-\tau,j) + \hat{\iota_b}(t-\tau,j)] \tag{S1b}$$

where $\hat{\iota_v}(t,j) = \sum_{s=1}^{t} i_v(t,j,s)$ and $\hat{\iota_b}(t,j) = \sum_{s=1}^{t} i_b(t,j,s)$ are the total number of individuals of age group j who have received a second shot or booster vaccination, respectively, and become infected on day t. Additionally, P_τ ($1 \leq \tau \leq \tau_{max}$, here τ_{max} = 14 days) is the generation time probability density function, and β(t) is the time-varying transmission matrix whose *ij*th element denotes the transmission rate between an individual from age-group *j* and individuals of age-group *i*; See below for details on the estimation of the generation-time distribution and the construction of the transmission matrix.

Similarly, the daily incidence of infections of vaccinated individuals in age group *j* on day t is given by and

$$i_v(t,j,s) = [1 - VE_v(s)]V(t,j,s)F_j(t),$$

$$i_b(t,j,s) = [1 - VE_b(s)]B(t,j,s)F_j(t), \qquad s = 1,2,\cdots,t \tag{S1c}$$

where $VE_V(s)$ and $VE_b(s)$ are the profiles of vaccine protection against infection of individuals s days after first dose vaccination or booster vaccination, respectively.

The simulation progresses day by day, $t = t_0, t_0 + 1, \ldots$, where t_0 is the start time of the simulation. (t_0= 194 in this work, corresponding to the time from the first vaccination in Israel to July 1,2021, the start date of our simulation). For each day *t* and group *j*, it performs the following steps:

1. Compute the daily incidence of infections, using (S1).
2. Update the number of susceptible individuals, taking into account infections and vaccinations with the first dose

   $$S(t,j) = S(t-1,j) - inv(t,j) - Tvac(t,j), \quad j = 1,\cdots,n$$

   where $T_{vac}(t, j)$ is the number of individuals of age group j who are vaccinated on day t.

3. Update number of vaccinated, taking into account infections, first dose vaccinations and booster vaccination

   $$V(t,j,s) = V(t-1,j,s-1) - iv(t,j,s-1) + \delta_{s=1}(s)Tvac(t,j) - Tboost(t,j,s), \qquad s = 1,\cdots,t$$

where $T_{boost}(t, j, s)$ is the number of individuals of age group j who were vaccinated s days before day t and received a booster vaccination which became effective on day t, and $\delta_{s=1}(s) = 1$ when $s = 1$, and 0 otherwise.

4. Update number individuals who have received the booster, taking into account infections, and new booster vaccinations

$$B(t,j,s) = B(t-1,j,s-1) - i_b(t,j,s-1) + \delta_{s=1}(s)\sum_{\tau=1}^{t} T_{\mathrm{boost}}(t,j,\tau), \quad s = 1,\cdots,t$$

Note that the above model neglects the possibility of reinfections.

Model parameters

Table S1 summarizes the list of model parameters and the basis for determining their values. In addition, the model inputs includes the transmission matrix and vaccination schedules. In what follows, we provide additional details on the parameters and inputs.

Generation time distribution The generation time distribution P_τ was set to a discretized version of a gamma distribution with a mean of 4 days and a standard deviation of 2.7 days, based on intervals between infections obtained from triplets of known infector-infectee/infector-infectee in the data set of confirmed cases in Israel during July-October 2021. A dataset collected by means of epidemiological investigations provides information on pairs of individuals of whom the first is the probable infector and the second is the infectee, as well as the probable date on which the infection occurred. This dataset contains a subset of 8687 triads of individuals of whom the second was infected by the first and the third was infected by the second. We used these transmission chains of size three in our data set and extracted the intervals between the date of infection of the second individual and the date of infection of the third individual (fig. S1). The dates of infection were reported by the infected individuals according to time of contact with known cases, so various biases are probable. We have therefore performed a sensitivity analysis to examine the effect of varying the assumed mean generation-time on our results (see below).

Transmission matrix The transmission matrix β is given by

$$\beta_{ij}(t) = \frac{R(t_0)}{\rho(M(t_0))} M_{ij}(t), \quad M_{ij}(t) = \begin{bmatrix} \delta_1 C_{11}(t) & \cdots & \delta_1 C_{1n}(t) \\ \vdots & \ddots & \vdots \\ \delta_n C_{n1}(t) & \cdots & \delta_n C_{nn}(t) \end{bmatrix}$$

where C is an age-group contact matrix, δ is a vector containing the relative susceptibilities of each age-group (set to 1 except for the young children age-group 0-9 whose relative susceptibility was obtained by fitting the data), $R(t_0)$ is the reproductive number at time $t = t_0$ (that is the reproductive number in the absence of immunity induced by vaccinations and previous infections, but with the effect of behavioral changes and testing that reduces transmission and curtails the natural generation time of the disease), and $\rho(M)$ denotes the spectral radius of a matrix M.

We modeled the contact matrix $C(t)$ as follows:

$$C_{ij}(t) = \omega_h \alpha_h(t) {F_{ij}}^h + \omega_w \alpha_w(t) {F_{ij}}^W + \omega_s \alpha_s(t) {F_{ij}}^S + \omega_c \alpha_c(t) {F_{ij}}^C$$

where F^h, F^w, F^s, F^c are household/work/school/community contact-frequency matrices, respectively, derived for Israel using census and survey data on key socio-demographic features [4] (fig. S2). The coefficients ω_h , ω_w , ω_s , ω_c express the number of contacts occurring in these different settings (relative to the household setting, so that $\omega_h = 1$). In [4] values for these parameters were inferred by fitting to the POLYMOD matrix [5]. We have found, however, that these parameter values do not provide an adequate fit to our data. We have therefore estimated these coefficients as part of our model calibration. The coefficients $\alpha_h(t)$, $\alpha_w(t)$, $\alpha_s(t)$ and $\alpha_c(t)$ describe the time variation of intensity contacts within the different settings to the overall contacts at time t. These are obtained from Google's COVID-19 community mobility report for Israel during the modeled period [22]. The school coefficients were determined according to the assessed level of operation of formal and informal educational institutions during the modeled period. In particular, this period included both summer vacation during July-August and Jewish holidays during September (fig. S3).

Vaccination and booster schedules. The vaccination schedule $T_{vac}(t, j)$ includes age-stratified first dose vaccine uptakes at daily resolution, and is acquired from the Israel Ministry of Health's database. Similarly, we acquired from this database the booster schedule $T_{boost}(t, j)$ that describes the number of individuals of age group j who received a booster vaccination on day *t*. Fig. S4 presents the booster uptake in Israel. The model requires a more detailed schedule $T_{boost}(t, j, s)$ that also contains the number of days *s* from the day of receiving the first vaccine shot to the day of receiving the booster. However, the organization of Israel Ministry of Health database makes it cumbersome to extract the distribution of days *s* since first dose vaccination for those who received a booster vaccination on day *t*. We therefore assumed that those who first who received a first dose of vaccine are also the first to receive a booster vaccine, and constructed the detailed schedule $T_{boost}(t, j, s)$ accordingly.

Both vaccination schedules are processed to account for vaccination of recovered individuals who were not detected. Particularly, we estimated the number of recovered undetected cases per age groups using the detection rate ρ_j , and subtracted them from the vaccination numbers in the vaccination schedule.

We verified that the model outcomes are insensitive to the assumptions above.

Vaccine efficacy profiles. We relied on estimates for vaccine efficacy against infection acquired in a large integrated health system in the USA [20], together with recent estimates acquired in a nationwide study from Israel which extends [4] to account for data collected in July-September 2021 (fig. S5A). We assumed the same vaccine efficacy waning profile for all ages, in accordance with studies showing that the efficacy of mRNA vaccines in reducing infections is nearly independent of age [1, 4, 7, 20, 24]. In view of the lack of data on vaccine efficacy in the long term, we further assumed that, in accordance with the trend line fitted in fig. S5A, vaccine protection against infection decays to zero roughly 300 days after the vaccination. For the period of this study, model outcomes were insensitive to this assumption.

The short-term booster protection profile estimated in a nationwide study from Israel [12] is used to extract the efficacy profile $VE_b(s)$ for individuals s days after receiving the booster for $s \leq 35$. During the first 10 days of this period, the efficacy profile $VE_b(s)$ strongly depends on the protection provided by the second dose of vaccine at the time of booster administration. For simplicity, we considered an averaged second-dose vaccine protection computed as if the booster shot is administered 200 days after the first dose was administered. This approximation is justified by the fact that both vaccine and booster uptakes spanned relatively short periods of time. We verified that results were insensitive to the estimated averaged value of second-dose vaccine protection at the time of booster administration. In the absence of data supporting quantitative estimates, we assumed that the

waning rate of booster protection from infection is half the waning rate of the protection provided by second dose vaccine (fig. S6B). Our results are, however, insensitive to this assumption because booster efficacy remains high during the time span of the present study.

The model assumes that the probability $p_{v,s}$ of a vaccinated individual to develop severe disease, *conditional* upon being infected, does not change with time since vaccination. Denoting vaccine efficacy in protecting from infection, in dependence on time t from second vaccination, by $VE_V(t)$ and in protection from severe disease by $VE_S(t)$, we then have

$$1 - VE_S(t) = \left(1 - VE_V(t)\right) \cdot \frac{p_{v,s}}{p_{u,s}},$$

where $p_{u,s}$ is the probability of an unvaccinated individual to develop severe disease, conditional on infection. Therefore

$$VE_S(t) = 1 - c(1 - VE_V(t)) \quad \text{(S2)}$$

where $c = \frac{p_{v,s}}{p_{u,s}}$. This allows us to project $VE_S(t)$ based on knowledge of $VE_V(t)$, once we estimate the single parameter c, requiring a single data point $VE_S(t_1)$:

$$c = \frac{1 - VE_s(t_1)}{1 - VE_v(t_1)}$$

In fig. S5B we estimate c by taking $t_1 = 185$ and $VE_S(t_1)$ as the mean of the estimates of vaccine efficacy against severe disease at t = 155, 185 and 215 from [4], and plot the corresponding linear profiles of vaccine efficacy against severe disease given by (S2), for age groups under and over 60. The good agreement of these profiles with the estimates of vaccine protection from severe disease from [4] (also plotted) despite the fact that only one parameter is fitted for each, lends support to the assumption that the waning of vaccine efficacy against severe disease is only due to the waning of protection against infection, and the conditional probability of developing severe outcomes for an infected case is fixed in time. Under the assumption of fixed detection rates, this also implies that the probability of a detected case to become severe is constant in time, but depends on age and vaccination status - we calculated these conditional probabilities using case data in Israel during July-October 2021, see parameters $\kappa_{nv,j}$, $\kappa_{v,j}$ and $\kappa_{b,j}$ in table S1.

Initialization

The initial values for the dynamic variables in the model were set as follows:

- $i_{nv,j}(t)$, $(t_0 - \tau_{max} \le t \le t_0 - 1)$ - set as the non-vaccinated detected cases from age-group j with a 4 days lag (mean detection time) divided by the reporting rate ρ_j (see below).
- $i_{v,j}(t)$, $(t_0 - \tau_{max} \le t \le t_0 - 1)$ - set as the vaccinated detected cases from age-group j with a 4 days lag divided by the reporting rate ρ_j .
- $S_j(t_0)$ - set as N_j minus the known non-vaccinated recovered cases up to time t_0 divided by the reporting rate, minus the vaccinated up to time t_0 and minus the infected at time t_0.

- $Vj(t_0)$ - set as the vaccinated from age-group j up to time t_0 minus those infected after vaccination up to time t_0.

Because the model was run starting from July 1st prior to the delivering of any booster shot, the variables B and i_b at time t_0 were set to zero for all age-groups.

Model calibration

Observation of infections The observation model connects the infection counts generated by the epidemic model to those observed in the data, taking into account the delay from infection to the identification of a case and the fact that only a fraction of infections are identified.

We denote by ρj the *reporting rates* for individuals of age group j, that is the probability that an infected case will be detected. We define the distribution of time from infection to detection by $\{d_\tau\}$, that is, d_τ is the probability that a person infected on day t will be detected on day $t + \tau$, assuming detection.

The expected numbers of non-vaccinated, vaccinated, and boosted individuals detected in each age group on day t are then given by:

$$\mu_{nv}(j,t) = \rho_j \sum_\tau d_\tau i_{nv}(t-\tau,j)\,,$$
$$\mu_v(j,t) = \rho_j \sum_\tau d_\tau i_v(t-\tau,j)$$
$$\mu_b(j,t) = \rho_j \sum_\tau d_\tau i_b(t-\tau,j)$$

Denoting by $\overline{\iota_{nv}}(t,j), \overline{\iota_v}(t,j), \overline{\iota_b}(t,j)$, the numbers of infected individuals of age group j detected among the non-vaccinated, vaccinated, and booster-vaccinated classes on day t, we assume

$$\overline{\iota_{nv}}(j,t) \sim NB(\mu = \mu_{nv}(j,t), r),$$
$$\overline{\iota_v}(j,t) \sim NB(\mu = \mu_v(j,t), r),$$
$$\overline{\iota_b}(j,t) \sim NB(\mu = \mu_b(j,t), r),$$

where μ is the mean of the negative binomial distribution and r is the dispersion parameter. The negative binomial distribution captures the variance in our data better than a Poisson model (which corresponds to $r \to \infty$). A negative binomial distribution can arise as a mixture of the Poisson distributions with means distributed as a gamma distribution- for example if the reporting rates on each day are drawn from a gamma distribution with the mean being ρj . It could also derive from various other sources of noise in our data that are not explicitly modeled.

The likelihood function. Under this observation model, the likelihood corresponding to the infection data, given an epidemic trajectory generated by the epidemic model is:

$$L = L_{nv} \cdot L_v \cdot L_b$$

where

$$L_{nv} = \prod_j \prod_t \frac{\Gamma(r+\overline{\iota_{nv}}(j,t))}{\Gamma(r)\cdot\overline{\iota_{nv}}(j,t)!} \cdot \left(\frac{r}{r+\mu_{nv}(j,t)}\right)^r \left(\frac{\mu_{nv}(j,t)}{r+\mu_{nv}(j,t)}\right)^{\overline{\iota_{nv}}(j,t)},$$

and similarly for L_v, L_b.

Because L depends on the epidemic model output for given parameter values, L is a function of these model parameters. The maximum likelihood estimates for the parameters are found by maximizing the function L, using numerical optimization. Overall, eight parameters were estimated as part of fitting the data (table S1): $R(t_0)$, the reproductive number at time t_0; ω_W,ω_S,ω_C, the intensities of contacts in the workplace, school, and community settings relative to that of household contacts; δ_1, the relative susceptibility of children age 0-9, ρ_1 , ρ_9 the reporting rates for individuals age 0-9 and individuals age 80+ (the reporting rates for the intermediate age groups are obtained by linear interpolation of these endpoints); and *r*, the dispersion parameter for the negative binomial distribution. Detailed results of the model fit are shown in fig. S7-S9. 95% CI for the parameter estimates were calculated using likelihood profiles [23] (fig. S13).

Modeling severe cases. We extended the model by modeling the flux of severe cases from detected cases. We extracted the incidence of severe cases by their confirmation time so that we do not need to model the time from detection to when severe illness began. The probability that an unvaccinated detected person of age-group j will become a severe case is denoted by $\kappa_{nv,j}$. Analogous probabilities for vaccinated individuals and individuals who have received a booster are denoted by $\kappa_{v,j}$ and $\kappa_{b,j}$. These probabilities were all extracted from the data (table S1). We assume that all severe cases are detected.

The expected numbers of unvaccincated, vaccinated and booster-vaccinated from age-group j detected at time t that will reach a severe state are then given by

$$sev_{nv}(t,j) = \kappa_{nv,j}\mu_{nv}(j,t), \quad sev_v(t,j) = \kappa_{v,j}\mu_v(j,t), \quad sev_b(t,j) = \kappa_{b,j}\mu_b(j,t).$$

Setting the parameters to the maximum likelihood estimates obtained from fitting the detected cases, we run the extended model and compare the results to the observed incidences of new severe cases. Detailed results of the model fit to these data are shown in fig. S10-S12.

Sensitivity Analysis. We performed a set of sensitivity analyses to test the robustness of results to the generation time distribution and detection time distribution employed in the model fitting procedure:

1. Instead of using a generation-time with mean of 4 days, we fixed the mean generation time to 3.5 days and 4.5 days (using a gamma distribution with the same s.d. of 2.7 days as before) and explored the effect on the results.
2. Instead of using mean detection time of 3.5 days, we fixed the mean detection time to 3 days and 4 days (using a gamma distribution with the same s.d. as before of 2.4 days) and explored the effect on the results.

The effect of these sensitivity analyses on the parameter estimates and the estimates of outcomes for the 'No-Boost' what-if scenario is given in table S2. While estimated parameter values vary somewhat when time distributions are varied, the estimates of outcomes

in the counterfactual no boost scenario, relative to the actual outcome, do not change to an extent that affects our general conclusions regarding the effect of the booster campaign.

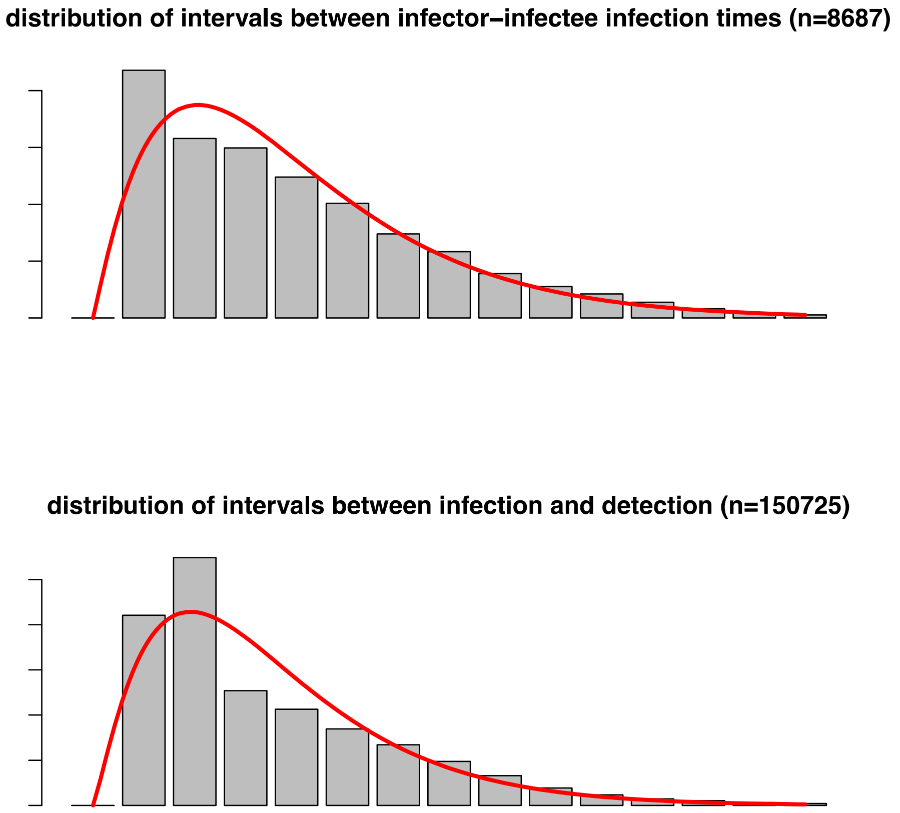

Figure S1: Distributions employed in the transmission and observation models. Top panel: The generation-time distribution. Bars show the empirical distribution of intervals between infections obtained from 8687 triplets of infector-infectee/infector-infectee in the data set of confirmed cases in Israel during July-October 2021. The red line shows the maximum-likelihood fit of a gamma distribution density function to the empirical data. Bottom panel: Detection time distribution. Bars show intervals of time between infection and detection for confirmed cases in Israel during July-October 2021 with an estimated infection date. The red line shows the maximum-likelihood fit of a gamma distribution density function to the empirical data.

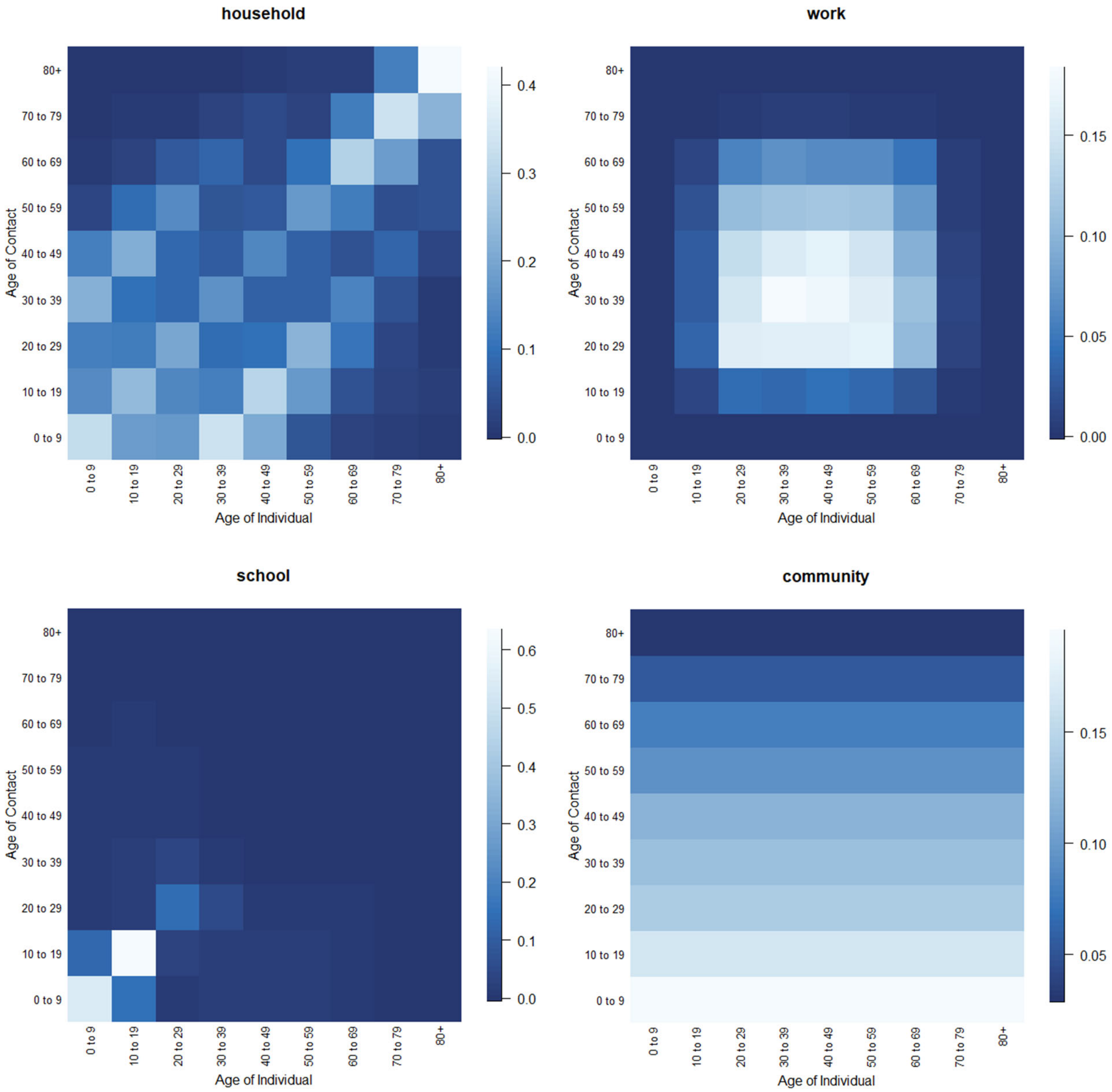

Figure S2: Contact-frequency matrices used in the fitting procedure. The matrices were taken from [4] and converted into 10-year bands instead of 5-year bands.

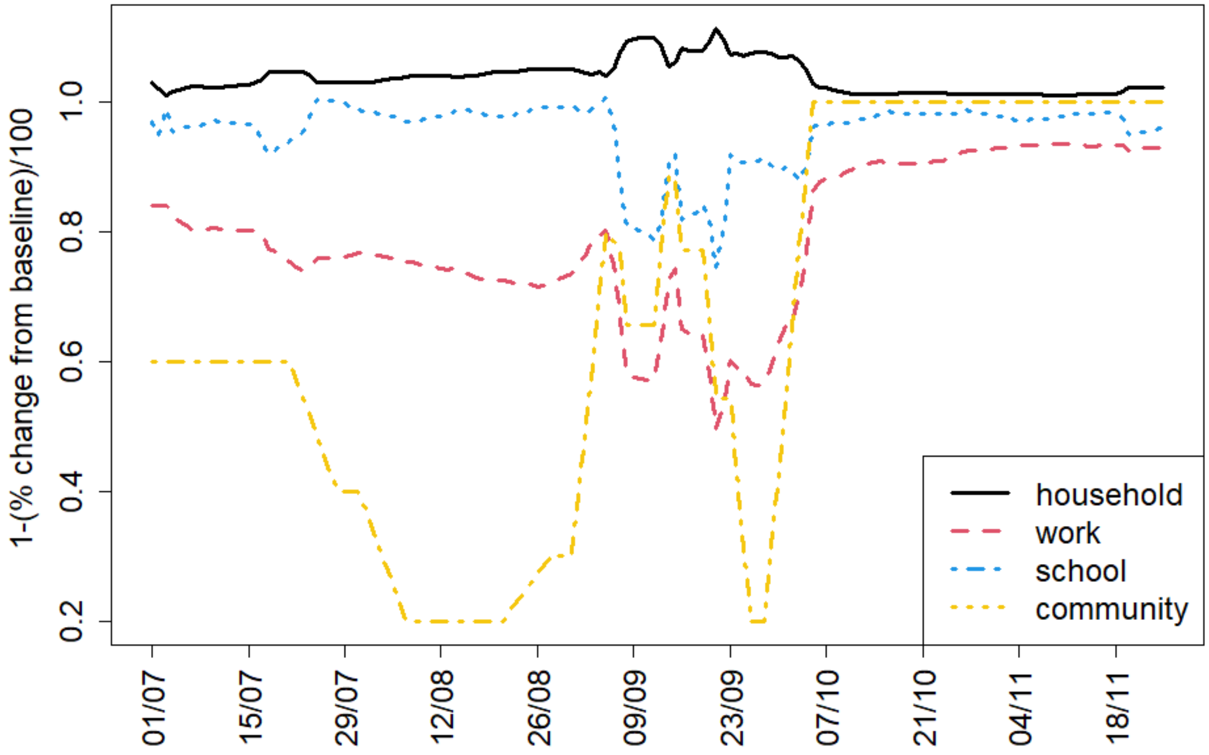

Figure S3: Daily coefficients for the contact matrices used in the fitting procedure. The *household* coefficient is based on Google Mobility Report *residential percent change from baseline*. The *work* coefficient is based on Google Mobility Report's *workplaces percent change from baseline*. The *community* coefficient is based on Google Mobility Report's *retail and recreation percent change from baseline*. The *school* coefficient was set according to the assessed proportion of school openings at each period. All curves were smoothed using a 7-day moving-average.

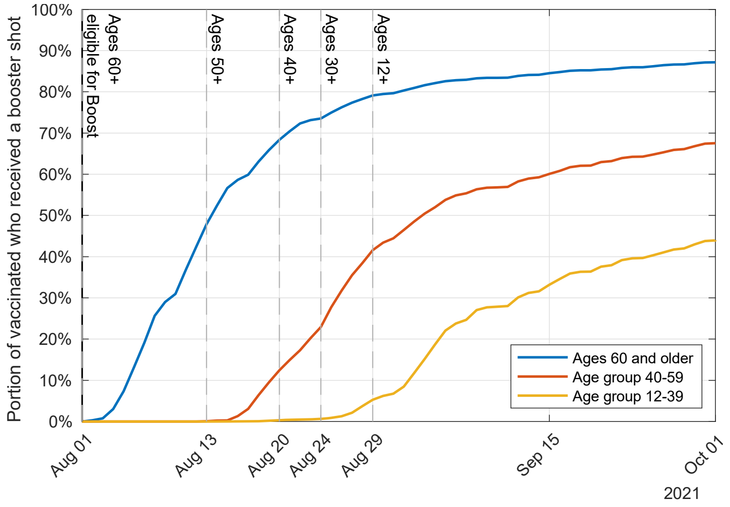

Figure S4: Booster uptake in Israel. The cumulative portion of the vaccinated population in each age group who received a booster shot.

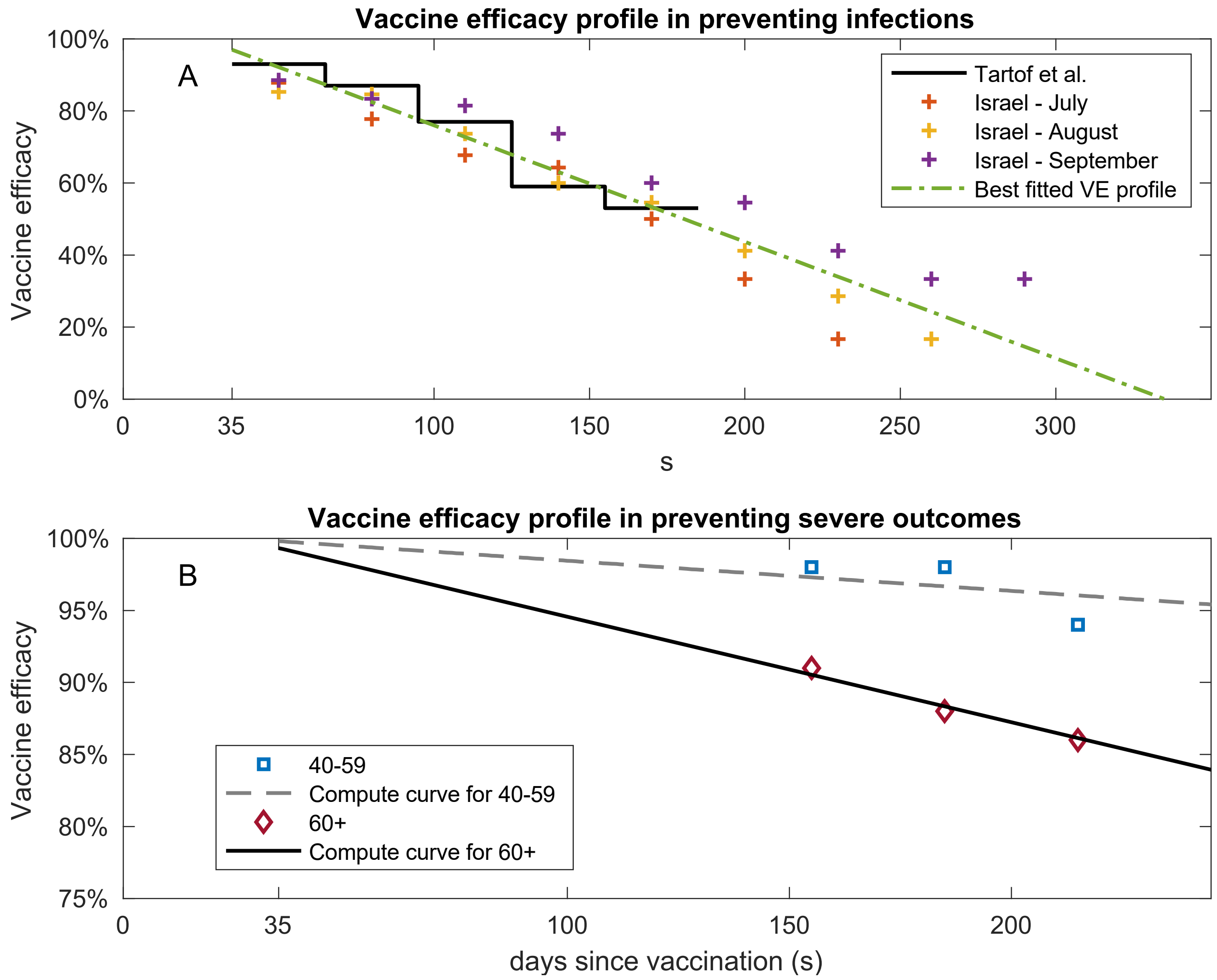

Figure S5: Estimates of vaccine waning profile. A: Estimates of vaccine efficacy profile in preventing infections [20] (Black solid curve), Extension of [4] to account for data collected in July-September 2021 (Cross markers). Superimposed is the best fitted vaccine efficacy profile. **B**: Estimates of vaccine efficacy against severe outcomes as a function of days after vaccination [4] for ages 40-59 (square blue markers) and ages 60 and older (diamond red markers). Superimposed are the best fitted vaccine efficacy profiles per age groups under the assumption that the protection against severe induced by vaccine, conditional on infection, does not vary with time since vaccination. In all cases, estimates start from time of full vaccination at day s = 35 after receiving the first shot.

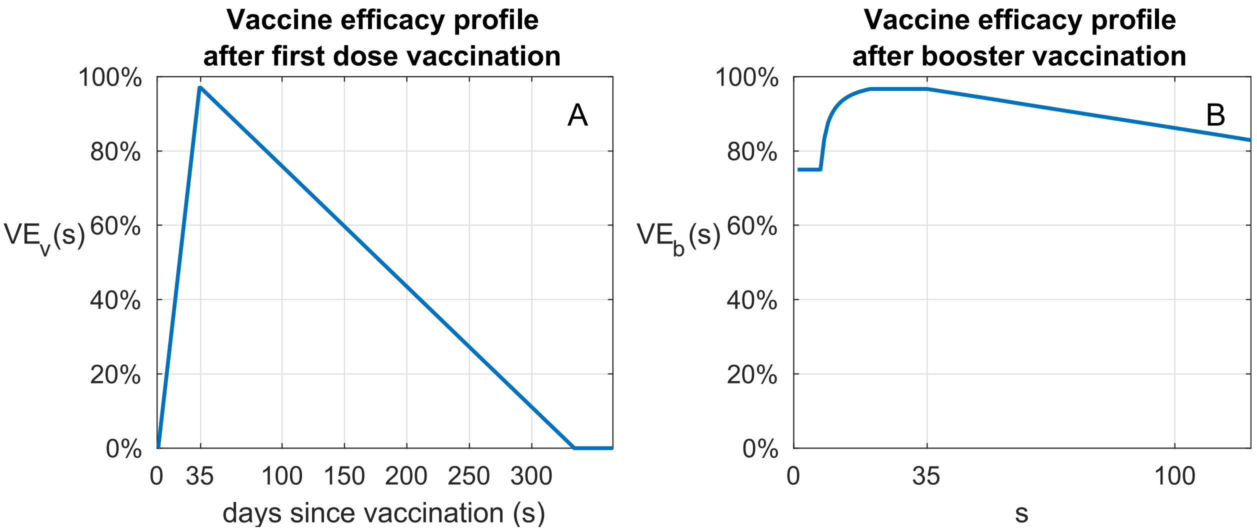

Figure S6: Vaccine efficacy profile from administration of first dose. A: Vaccine efficacy profile $VE_v(s)$ of individuals s days after receiving the first dose of vaccination. B: Vaccine efficacy profile $VE_b(s)$ of individuals s days after receiving booster vaccination.

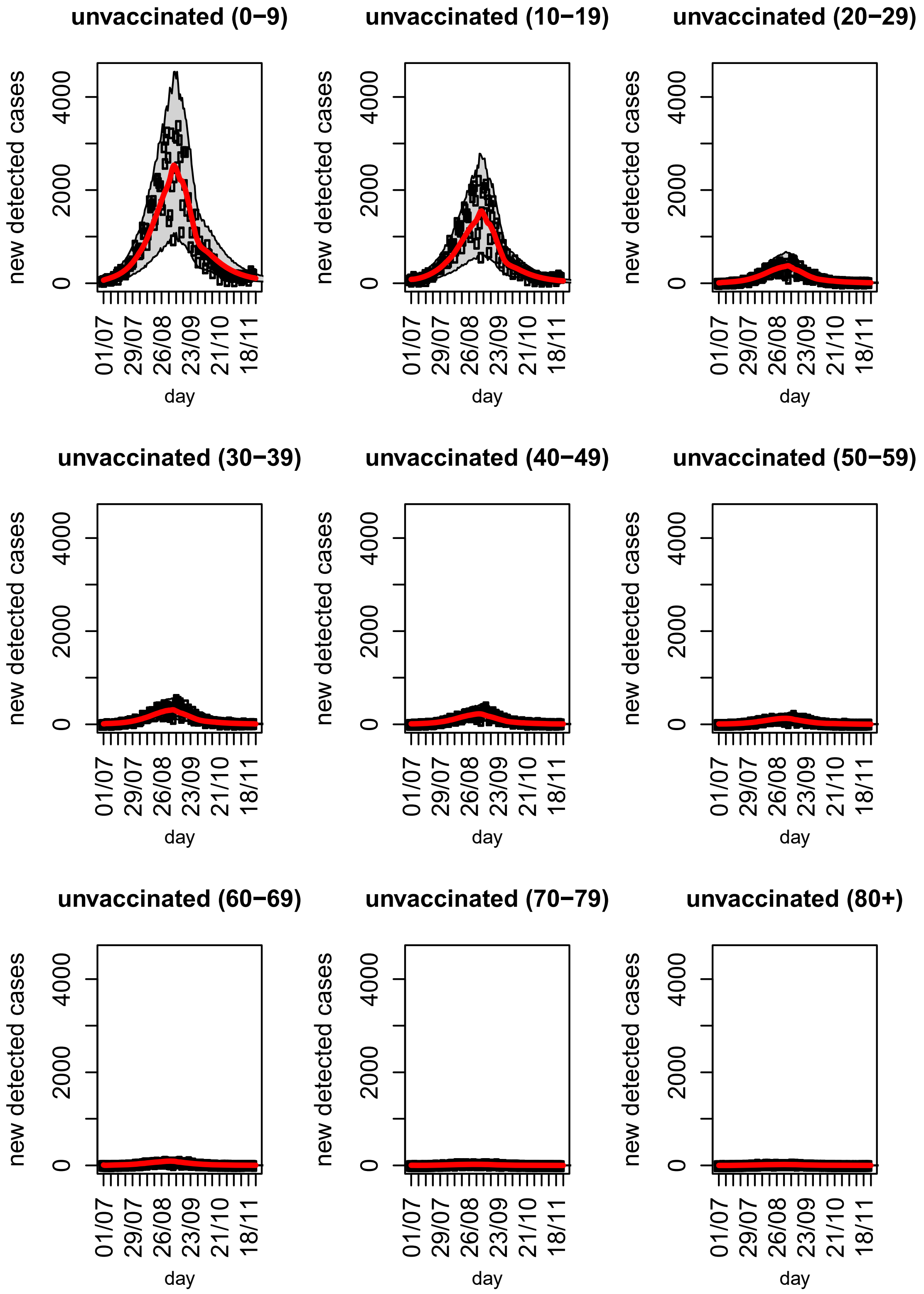

Figure S7: Model fit to detected cases incidences in unvaccinated individuals. Model fit (red curves) in comparison to the observed data (black markers). The grey area in each graph indicates 95% predictive intervals obtained from 1000 simulations using the negative binomial distribution with the estimated dispersion parameter.

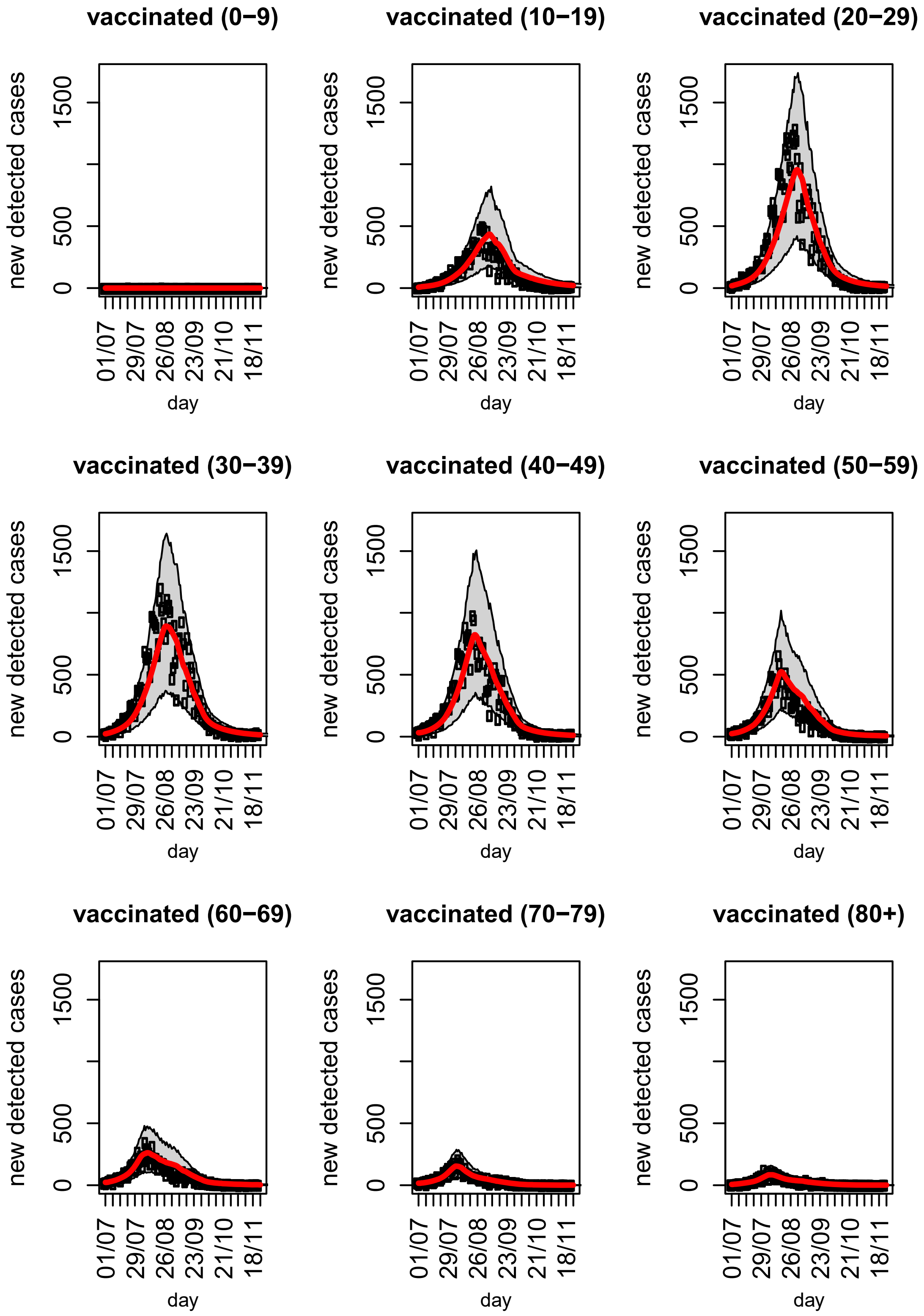

Figure S8: Model fit to detected cases incidences in vaccinated individuals. Model fit (red curves) in comparison to the observed data (black markers). The grey area in each graph indicates 95% predictive intervals obtained from 1000 simulations using the negative binomial distribution with the estimated dispersion parameter.

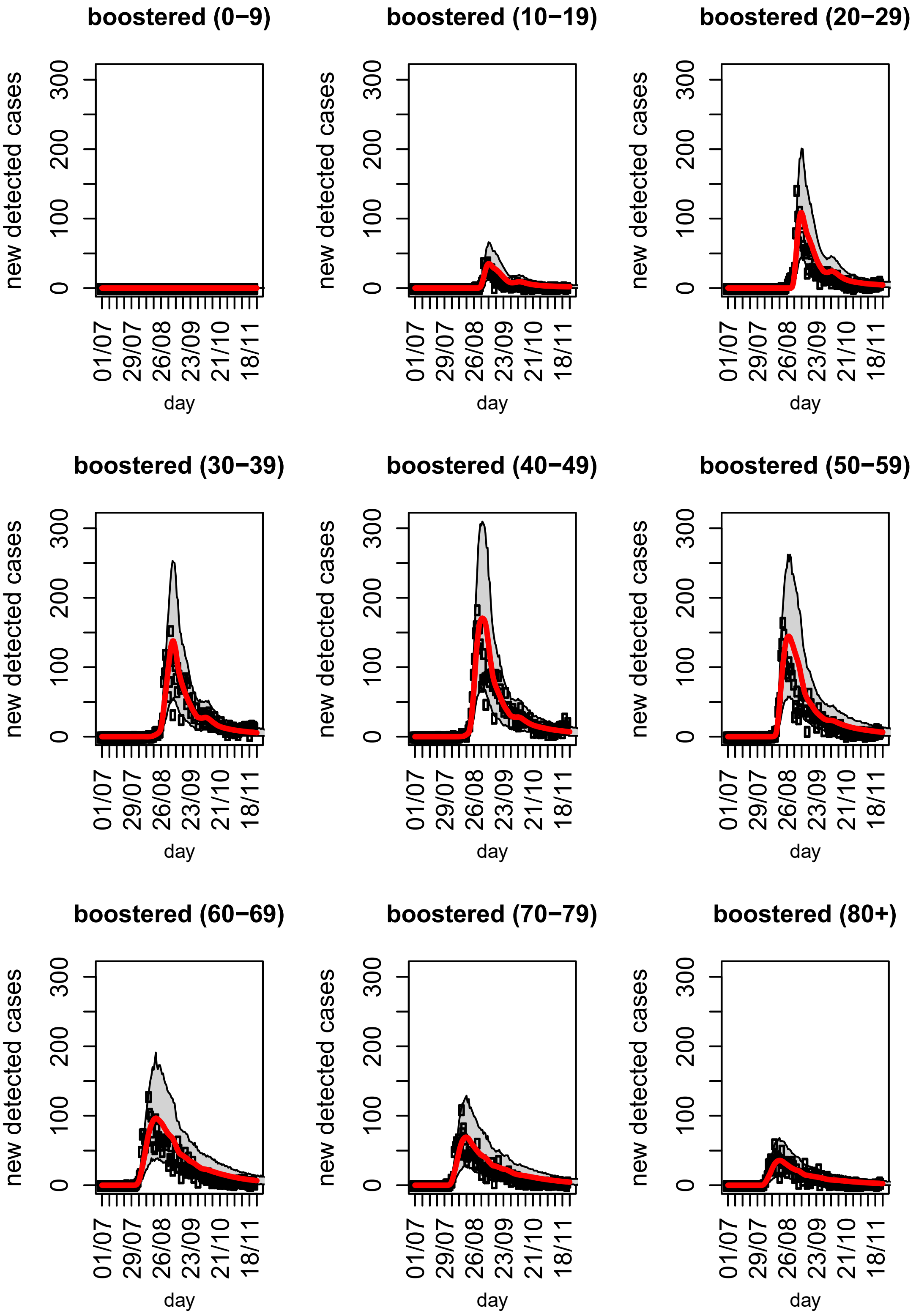

Figure S9: Model fit to detected cases incidences in boostered individuals. Model fit (red curves) in comparison to the observed data (black markers). The grey area in each graph indicates 95% predictive intervals obtained from 1000 simulations using the negative binomial distribution with the estimated dispersion parameter.

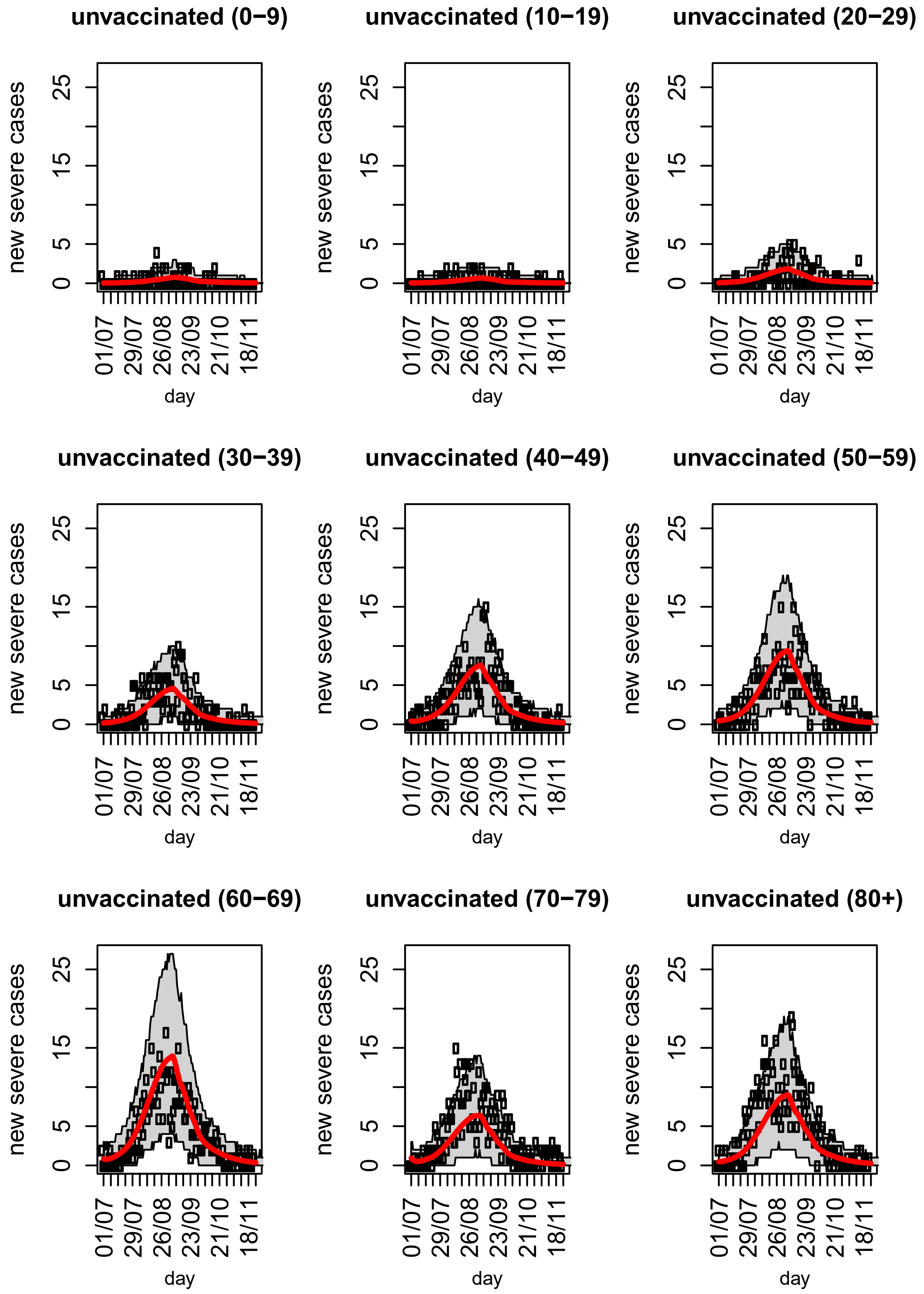

Figure S10: Model fit to severe cases incidences in unvaccinated individuals. Model fit (red curves) in comparison to the observed data (black markers). The grey area in each graph indicates 95% predictive intervals obtained from 1000 simulations using the negative binomial distribution with the estimated dispersion parameter.

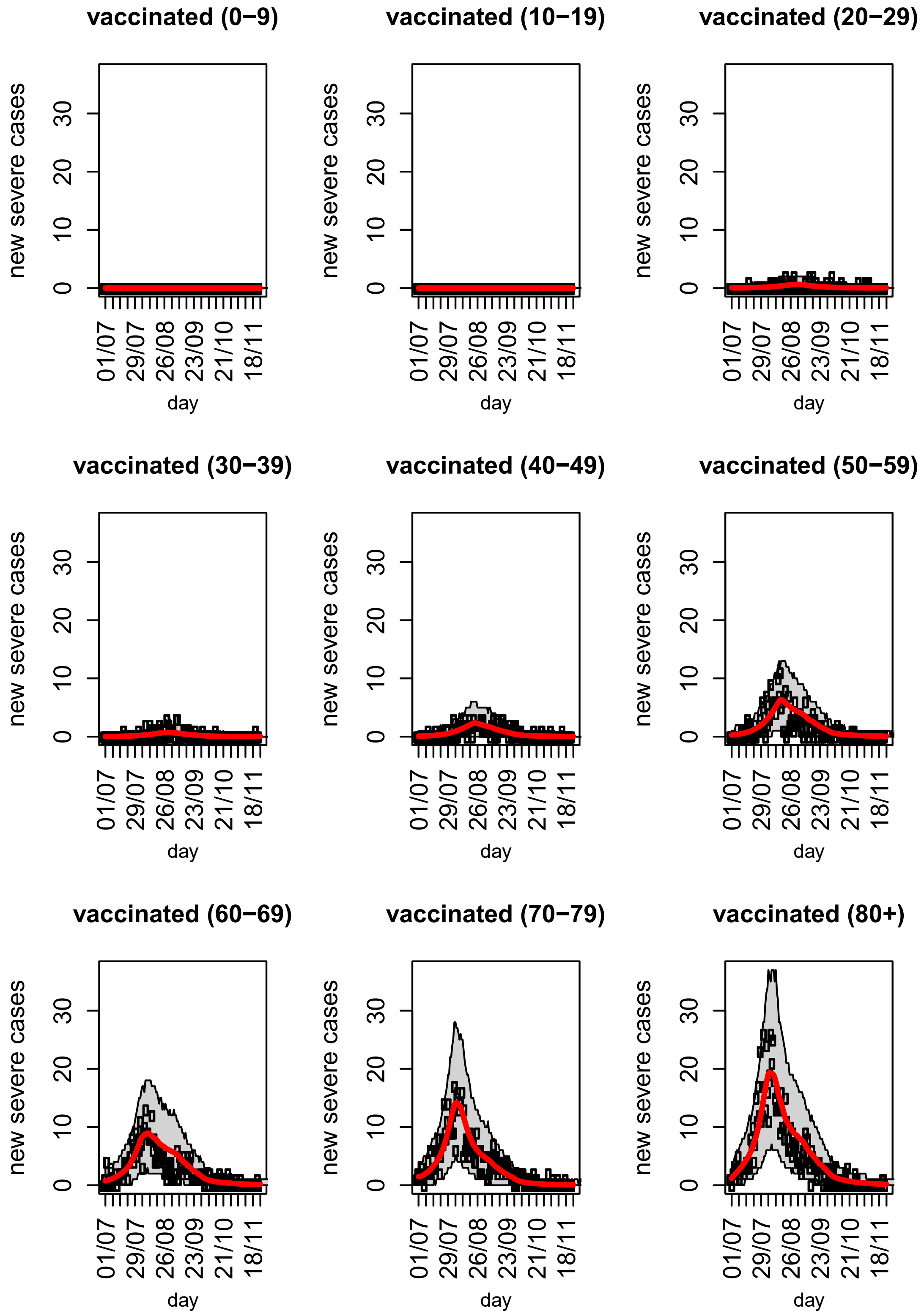

Figure S11: Model fit to severe cases incidences in vaccinated individuals. Model fit (red curves) in comparison to the observed data (black markers). The grey area in each graph indicates 95% predictive intervals obtained from 1000 simulations using the negative binomial distribution with the estimated dispersion parameter.

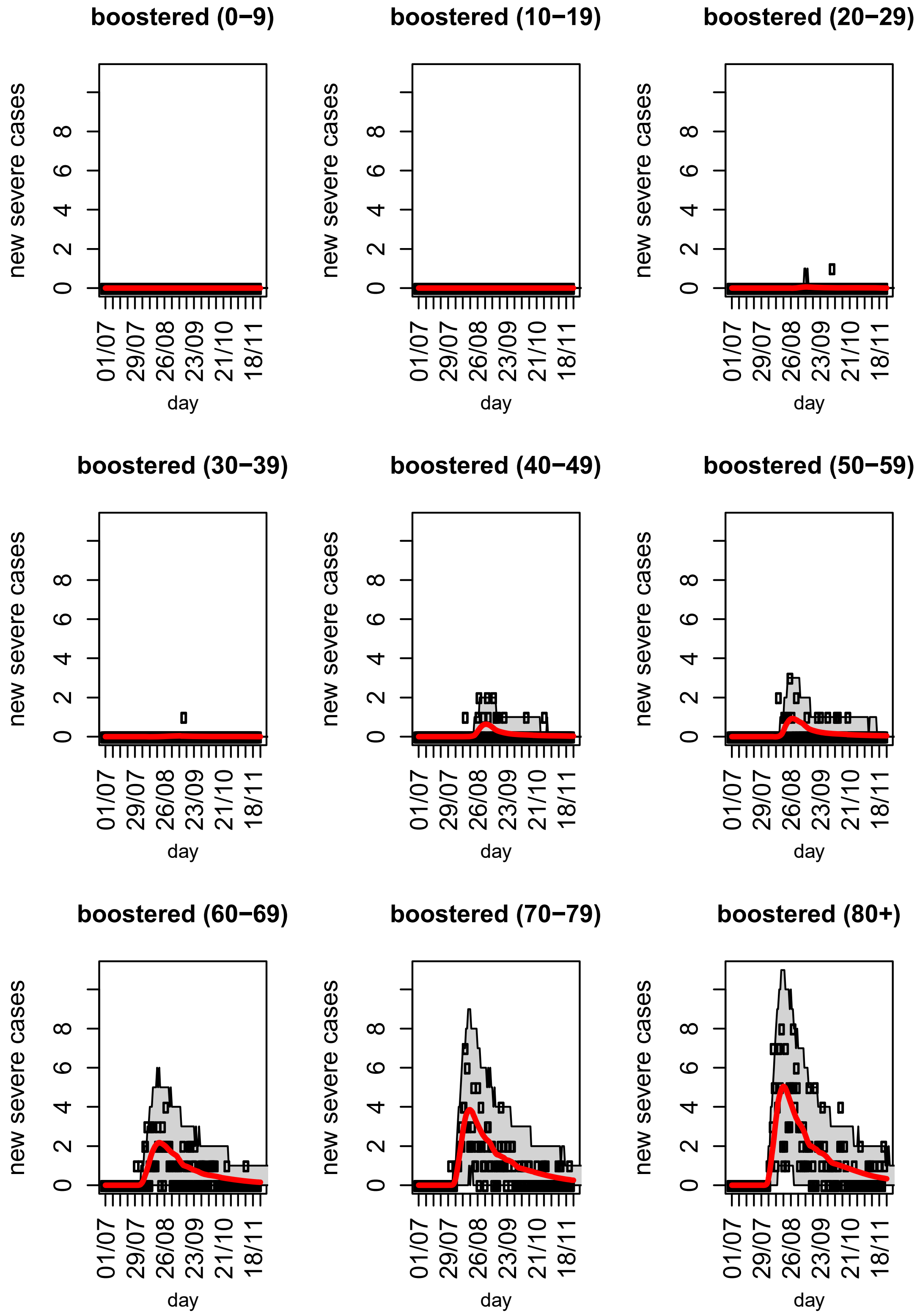

Figure S12: Model fit to severe cases incidences in boostered individuals. Model fit (red curves) in comparison to the observed data (black markers). The grey area in each graph indicates 95% predictive intervals obtained from 1000 simulations using the negative binomial distribution with the estimated dispersion parameter.

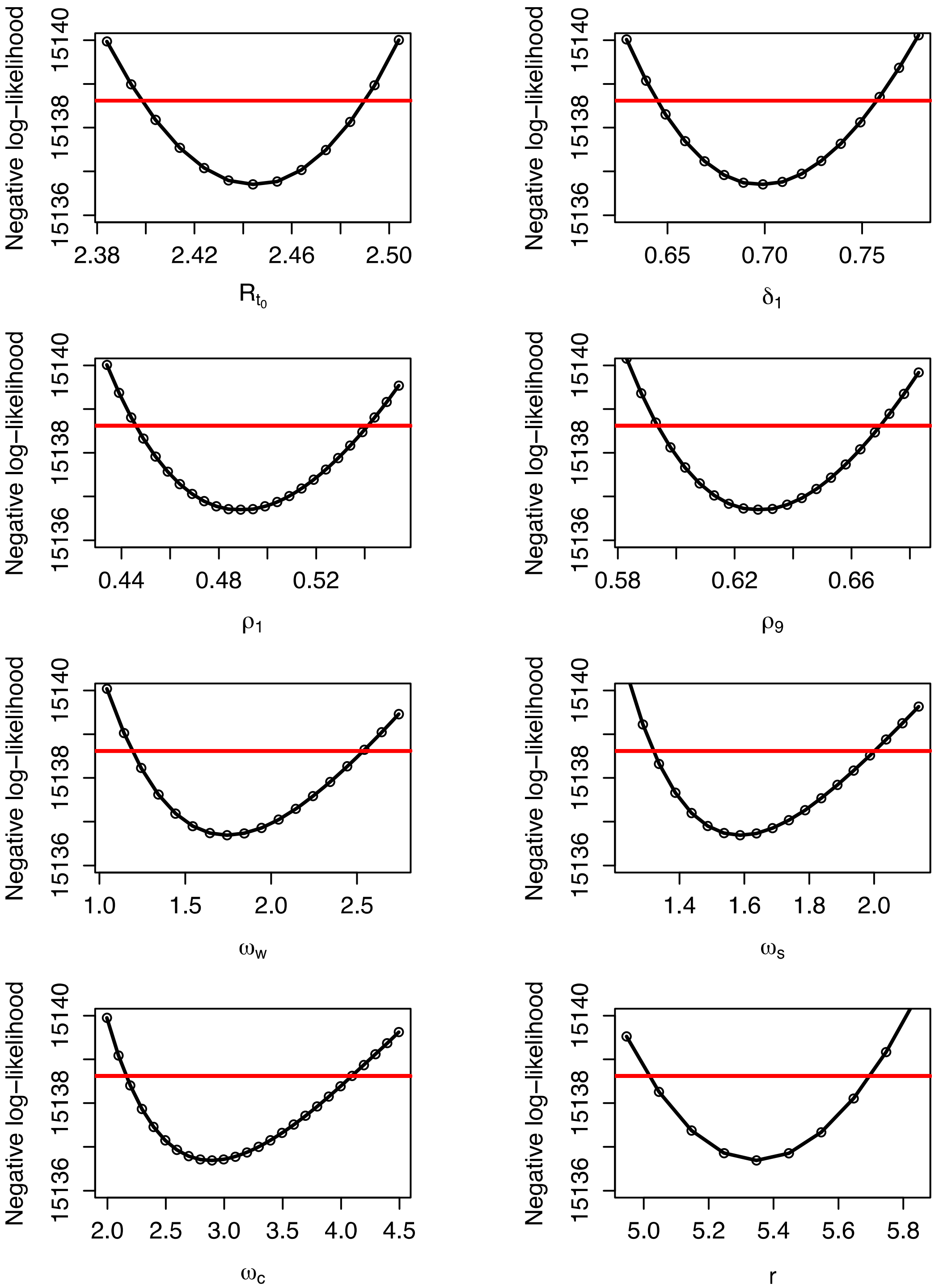

Figure S13: Likelihood profiles for the estimated parameters. The 95% CI for the parameter estimates that are presented in table S1 were taken as the values whose log-likelihood are within 1.92 from the maximum likelihood value (threshold marked by the red line).

Table S1: Model parameters.

Parameter	Meaning	Value	Basis for value set
N_i	Age-group sizes	1830,1550,1310,1210,1106, 864,747,500,283 (1e3)	Israel Central Bureau of Statistics
P_τ	Generation-time distribution	~ Gamma with mean of 4 days and s.d. of 2.7 days	Estimated using contact-tracing data in Israel during July-October 2021 (see top panel in Figure S1)
d_τ	Time distribution from infection to detection	~ Gamma with mean of 3.5 days and s.d. of 2.4 days	Estimated using contact-tracing data in Israel during July-October 2021 (see bottom panel in Figure S1)
$VE_v(s)$	Profile of vaccine protection against infection for individuals *s* days after first dose vaccination	See fig. S6A	Relies on the vaccine efficacy estimates presented in [1, 2].
$VE_b(s)$	Profile of vaccine protection against infection for individuals *s* days after booster vaccination	See fig. S6B	Relies on the vaccine efficacy estimates presented in [3].
$R(t_0)$	The reproductive number at time $t_0 = 194$ (July 1, 2021)	2.44 [2.40-2.49]	Estimated by fitting the data
δ_j	relative susceptibility of individuals of age-group *j*	$\delta_1 = 0.70$ [0.64-0.76] , $\delta_{j>1} = 1$	δ_1 was estimated by fitting the data, while $\delta_{j>1}$ were fixed to 1
ρ_j	Probability of an infected case of age-group *j* to be detected	$\rho_1 = 0.49$ [0.44-0.54], $\rho_9 = 0.63$ [0.59-0.67]	Estimated by fitting the data using 2 parameters: one for individuals age 0-9 and one for individuals age 80+. Rates for the intermediate age groups are obtained using linear interpolation of these two endpoints.
ω_w<br>ω_s<br>ω_c	Number of contacts occurring within the workplace, school and community settings (relative to the household setting, so *that* $\omega_h = 1$)	$\omega_w = 1.7$ [1.1-2.5]<br>$\omega_s = 1.6$ [1.3-2.0]<br>$\omega_c = 2.9$ [2.1-4.1]	Estimated by fitting the data
r	Dispersion parameter of the negative binomial distribution used in the observation model	5.3 [5.0-5.7]	Estimated by fitting the data
$\kappa_{nv,j}$	Probability of an unvaccinated detected case of age-group *j* to become a severe case	0.0003, 0.0004, 0.0049, 0.0148, 0.0335, 0.0723, 0.1537, 0.2687, 0.4635	Fraction of unvaccinated detected cases who developed severe illness in Israel during July-November 2021

$\kappa_{v,j}$	Probability of a vaccinated detected case of age-group *j* to become a severe case	0.0000, 0.0000, 0.0007, 0.0008, 0.0028, 0.0121, 0.0342, 0.0920, 0.2304	Fraction of vaccinated detected cases who developed severe illness in Israel during July-November 2021
$\kappa_{b,j}$	Probability of a boostered detected case of age-group *j* to become a severe case	0.0000, 0.0000, 0.0005, 0.0003, 0.0037, 0.0064, 0.0225, 0.0558, 0.1415	Fraction of boostered detected cases who developed severe illness in Israel during July-November 2021
η_j	Probability of mortality for a severe case of age-group j	0.02, 0.00, 0.05, 0.07, 0.08, 0.14, 0.30, 0.37, 0.50	Fraction of severe cases who died in Israel during July-November 2021 (no significant difference related to vaccination status was found)

Table S2: Results of sensitivity analysis. Comparison of parameter estimates and model outputs using the main setting in fitting the data and alternative settings. The first five rows show the effect on the estimated model parameters. The next three rows show the effect on the model estimates for the 'No Boost' what-if scenario, which is presented in the main text in Table 1.

output	main	mean(P_τ)=3.5	mean(P_τ)=4.5	mean(d_τ)=3	mean(d_τ)=4
**R(t0)**	2.44	2.33	2.58	2.42	2.47
**δ1**	0.70	0.72	0.68	0.70	0.69
**ρ1**	0.49	0.53	0.46	0.50	0.47
**ρ9**	0.63	0.71	0.55	0.64	0.61
**ωw**	1.7	1.3	2.8	1.7	1.9
**ωs**	1.6	1.3	2.3	1.5	1.7
**ωc**	2.9	2.2	4.5	2.9	2.9
**r**	5.3	5.3	5.2	5.3	5.3
**No Boost: change in cases**	501%	540%	469%	517%	481%
**No Boost: change in severe cases**	904%	991%	828%	935%	866%
**No Boost: change in mortality**	1086%	1195%	992%	1124%	1040%
