# Supplementary material for: Population-level implications of the Israeli booster campaign to curtail COVID-19 resurgence
Source: Sci Transl Med. 2022 Apr 12:eabn9836. doi: 10.1126/scitranslmed.abn9836 (PMC9012104; doi:10.1126/scitranslmed.abn9836)
Supplement: Supplementary file 2 — MDAR Reproducibility Checklist [file scitranslmed.abn9836_reproducibility_checklist.pdf]

Materials Design Analysis Reporting (MDAR) Checklist for Authors

The MDAR framework establishes a minimum set of requirements in transparent reporting applicable to studies in the life sciences (see Statement of Task: doi:10.31222/osf.io/9sm4x.). The MDAR checklist is a tool for authors, editors, and others seeking to adopt the MDAR framework for transparent reporting in manuscripts and other outputs. Please refer to the MDAR Elaboration Document for additional context for the MDAR framework.

For all that apply, please note where in the manuscript the required information is provided.

Materials:

Newly created materials	indicate where provided: section/legend	n/a
The manuscript includes a dedicated "materials availability statement" providing transparent disclosure about availability of newly created materials including details on how materials can be accessed and describing any restrictions on access.		x

Antibodies	indicate where provided: section/legend	n/a
For commercial reagents, provide supplier name, catalogue number and RRID, if available.		x

DNA and RNA sequences	indicate where provided: section/legend	n/a
**Short novel DNA or RNA including primers, probes:** Sequences should be included or deposited in a public repository.		x

Cell materials	indicate where provided: section/legend	n/a
**Cell lines:** Provide species information, strain. Provide accession number in repository **OR** supplier name, catalog number, clone number, **OR** RRID.		x
**Primary cultures:** Provide species, strain, sex of origin, genetic modification status.		x

Experimental animals	indicate where provided: section/legend	n/a
**Laboratory animals or Model organisms:** Provide species, strain, sex, age, genetic modification status. Provide accession number in repository **OR** supplier name, catalog number, clone number, **OR** RRID.		x
**Animal observed in or captured from the field:** Provide species, sex, and age where possible.		x

Plants and microbes	indicate where provided: section/legend	n/a
**Plants:** provide species and strain, ecotype and cultivar where relevant, unique accession number if available, and source (including location for collected wild specimens).		x
**Microbes:** provide species and strain, unique accession number if available, and source.		x

Human research participants	indicate where provided: section/legend; or state if these demographics were not collected	n/a
If collected and within the bounds of privacy constraints report on age, sex and gender or ethnicity for all study participants.	We have provided this information in a paragraph titled ‘Data:’ in Section 2 of the main text.	

Design:

**Study protocol**	**indicate where provided: section/legend**	**n/a**
If study protocol has been pre-registered, provide DOI. For clinical trials, provide the trial registration number **OR** cite DOI.		x

**Laboratory protocol**	**indicate where provided: section/legend**	**n/a**
Provide DOI **OR** other citation details if detailed step-by-step protocols are available.		x

**Experimental study design (statistics details)**		
**For in vivo studies:** State whether and how the following have been done	**indicate where provided: section/legend. If it could have been done, but was not, write not done**	**n/a**
Sample size determination		x
Randomisation		x
Blinding		x
Inclusion/exclusion criteria		x

**Sample definition and in-laboratory replication**	**indicate where provided: section/legend**	**n/a**
State number of times the experiment was replicated in laboratory.		x
Define whether data describe technical or biological replicates.		x

**Ethics**	**indicate where provided: section/legend**	**n/a**
**Studies involving human participants:** State details of authority granting ethics approval (IRB or equivalent committee(s), provide reference number for approval.	Ethics statement: The study was approved by the Institutional Review Board of the Sheba Medical Center. Helsinki approval number: SMC-8228-21.	
**Studies involving experimental animals:** State details of authority granting ethics approval (IRB or equivalent committee(s), provide reference number for approval.		x
**Studies involving specimen and field samples:** State if relevant permits obtained, provide details of authority approving study; if none were required, explain why.		x

**Dual Use Research of Concern (DURC)**	**indicate where provided: section/legend**	**n/a**
If study is subject to dual use research of concern regulations, state the authority granting approval and reference number for the regulatory approval.		x

Analysis:

Attrition	indicate where provided: section/legend	n/a
Describe whether exclusion criteria were preestablished. Report if sample or data points were omitted from analysis. If yes report if this was due to attrition or intentional exclusion and provide justification.		x

Statistics	indicate where provided: section/legend	n/a
Describe statistical tests used and justify choice of tests.		x

Data availability	indicate where provided: section/legend	n/a
For newly created and reused datasets, the manuscript includes a data availability statement that provides details for access or notes restrictions on access.	A data availability statement is provided at end of main text.	
If newly created datasets are publicly available, provide accession number in repository **OR** DOI **OR** URL and licensing details where available.	A (Zenodo) DOI to a repository is provided within the data availability statement	
If reused data is publicly available provide accession number in repository **OR** DOI **OR** URL, **OR** citation.	A (Zenodo) DOI to a repository is provided within the data availability statement	

Code availability	indicate where provided: section/legend	n/a
For all newly generated custom computer code/software/mathematical algorithm or re-used code essential for replicating the main findings of the study, the manuscript includes a data availability statement that provides details for access or notes restrictions.	A data availability statement is provided at end of main text.	
If newly generated code is publicly available, provide accession number in repository, **OR** DOI **OR** URL and licensing details where available. State any restrictions on code availability or accessibility.	A (Zenodo) DOI to a github repository is provided within the data availability statement	
If reused code is publicly available provide accession number in repository **OR** DOI **OR** URL, **OR** citation.		x

Reporting

MDAR framework recommends adoption of discipline-specific guidelines, established and endorsed through community initiatives. Journals have their own policy about requiring specific guidelines and recommendations to complement MDAR.

**Adherence to community standards**	**indicate where provided: section/legend**	**n/a**
State if relevant guidelines (e.g., ICMJE, MIBBI, ARRIVE) have been followed, and whether a checklist (e.g., CONSORT, PRISMA, ARRIVE) is provided with the manuscript.		x
